# A massacre of early Neolithic farmers in the high Pyrenees at Els Trocs, Spain

**DOI:** 10.1038/s41598-020-58483-9

**Published:** 2020-02-07

**Authors:** Kurt W. Alt, Cristina Tejedor Rodríguez, Nicole Nicklisch, David Roth, Anna Szécsényi Nagy, Corina Knipper, Susanne Lindauer, Petra Held, Íñigo García Martínez de Lagrán, Georg Schulz, Thomas Schuerch, Florian Thieringer, Philipp Brantner, Guido Brandt, Nicole Israel, Héctor Arcusa Magallón, Christian Meyer, Balazs G. Mende, Frieder Enzmann, Veit Dresely, Frank Ramsthaler, José Ignacio Royo Guillén, Eva Scheurer, Esther López Montalvo, Rafael Garrido Pena, Sandra L. Pichler, Manuel A. Rojo Guerra

**Affiliations:** 1 Center of Natural and Cultural Human History, Danube Private University, Krems, Austria; 2 Department of Biomedical Engineering, University of Basel, Basel, Switzerland; 3 Integrative Prehistory and Archaeological Science, University of Basel, Basel, Switzerland; 4 Juan de la Cierva-Formación Programme. Institute of Heritage Sciences, Spanish National Research Council (Incpit-CSIC) Spain, Valladolid, Spain; 5 State Office for Heritage Management and Archaeology, Halle State Museum of Prehistory, Halle, Germany; 6 Institute of Archaeology, Research Centre for the Humanities, Hungarian Academy of Sciences, Budapest, Hungary; 7 Curt-Engelhorn-Zentrum Archaeometrie gGmbH, Mannheim, Germany; 8 Juan de la Cierva-Incorporación Programme, Department of Prehistory and Archaeology, Faculty of Philosophy and Letters, University of Valladolid, Valladolid, Spain; 9 Max Planck Institute for the Science of Human History, Jena, Germany; 10 Friedrich-Loeffler-Institute for Medical Microbiology, University of Greifswald, Greifswald, Germany; 11 Arcadia-General Foundation of Valladolid University, Valladolid, Spain; 12 OsteoARC - OsteoArchaeological Research Center, Goslar, Germany; 13 Computer Tomography Lab of the Institute of Hydrogeochemistry, University of Mainz, Mainz, Germany; 14 Institute of Forensic Medicine, University of Saarland, Homburg, Germany; 15 General Directorate of Culture and Heritage, Government of Aragon, Zaragoza, Spain; 16 Institute of Forensic Medicine, University of Basel, Basel, Switzerland; 17 Chargée de recherche CNRS, Laboratoire TRACES UMR 5608, Université de Toulouse II-Jean Jaurès, Toulouse, France; 18 Department of Prehistory and Archaeology, Faculty of Philosophy and Letters, Atonomous University of Madrid, Madrid, Spain; 19 Department of Prehistory and Archaeology, Faculty of Philosophy and Letters, Valladolid University, Valladolid, Spain

**Keywords:** Anthropology, Archaeology

## Abstract

Violence seems deeply rooted in human nature and an endemic potential for such is today frequently associated with differing ethnic, religious or socio-economic backgrounds. Ethnic nepotism is believed to be one of the main causes of inter-group violence in multi-ethnic societies. At the site of Els Trocs in the Spanish Pyrenees, rivalling groups of either migrating early farmers or farmers and indigenous hunter-gatherers collided violently around 5300 BCE. This clash apparently resulted in a massacre of the Els Trocs farmers. The overkill reaction was possibly triggered by xenophobia or massive disputes over resources or privileges. In the present, violence and xenophobia are controlled and sanctioned through social codes of conduct and institutions. So that, rather than representing an insurmountable evolutionary inheritance, violence and ethnic nepotism can be overcome and a sustainable future achieved through mutual respect, tolerance and openness to multi-ethnic societies.

## Introduction

It was once a widespread idea that humans, while they were hunter-gatherers, were by nature good and peaceful due to the existence of an “egalitarian ethic”^1^. After the introduction of farming 12,000 years ago, adaptive socio-cultural strategies, which went hand in hand with, inter alia, sedentism, hierarchies and property, would have radically broken with the original way of life. In order to defend property and possessions, humans had then assumedly been forced to use violence. The existence of a non-violent past is now considered disproved, as aggressive behaviour has generally been demonstrated in both prehistoric and modern hunter-gatherer communities^2,3^. The extent and role of violence in hunter-gatherer communities is, however, still debated^4^. Is violence therefore an evolutionary legacy? The basic fact seems obvious, because humans are not the only species of great ape that kill their conspecifics. Jane Goodall^5^ and others impressively documented in field research how aggression and conflicts characterize the everyday life of *Pan troglodytes*^6^. Evolutionary and behavioural approaches to research into violence assign to human aggression both significant phylogenetic and ontogenetic components, deeply rooted in human nature^7,8^. Violence is therefore a part of our history as a species and of our existence as individuals. This realisation is difficult to accept for people today, as we consider ourselves rational and equipped with mechanisms to control our actions (Supplementary Text S1).

**Figure 1:**
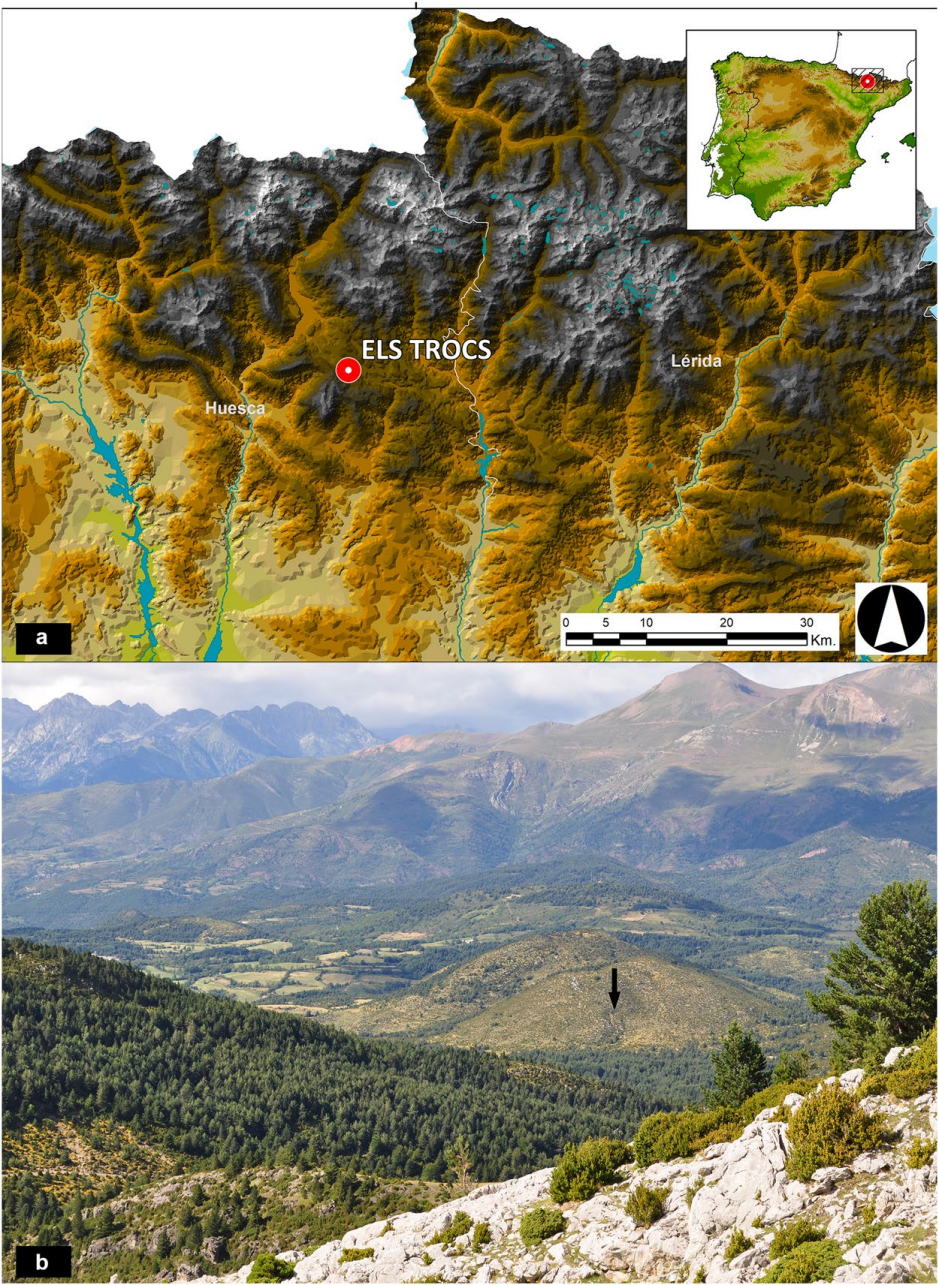
Geographic location of the Els Trocs cave site in Spain and the Pyrenees and its mountain setting on the *Selvaplana* plateau. (**a**) Location of the site and the two neighbouring northeastern Spanish provinces of Huesca and Lérida (Lleida) on a topographic map of the Pyrenees (map: ArGIS 10, license of University of Valladolid). (**b**) View of the Els Trocs cave entrance at 1,530 masl located on the southern slope of a karst hill on the high plateau of *Selvaplana; seen* from the pass of the Puerto de las Aras at 1,904 masl (photo: H. Arcusa Magallón).

This contribution was prompted by archaeological excavations in the Els Trocs cave (Sant Feliu de Veri, Bisaurri, Huesca) in the Spanish Pyrenees. At over 1500 m a.s.l., in the middle of a high plateau, a mountain rises up with the cave entrance on its slope (Fig. 1). In addition to material remains of former occupants such as ceramic and stone tools, it contains bones of butchered domestic and wild animals as well as human skeletal remains of children and adults. The 13 individuals identified so far can be assigned to three different Neolithic occupation phases that are far apart in time^9^. Thus, these “burials” are not one group who shared a single common fate. The following analysis focuses on nine individuals (five adults, four children) from the earliest occupation (phase I) of the cave 5,326–5,067 cal. BCE, whose radiocarbon dates cluster tightly and all of whom show traces of peri- and post-mortem violence (Table 1, Supplementary Text S2). The phase I individuals are thus distinct in being chronologically separated by over 1000 years from those in phase II as well as exhibiting a specific and unique set of taphonomically characteristic lesions (Table 2).

**Table 1:** Minimum number of individuals (ID: CET) from Els Trocs and two potential further individuals represented by isolated funnel-shaped cranial bone fragments (CET-F); Individual data on Age at death (in years) and Sex (morphologic/genetic; n.d. = not determined); Genetic profiles (^a^HVS1 data Mainz University, ^b^Genome-wide data Harvard University Boston and MPI Jena, ^c^Genome-wide data MPI Jena); occurrence of fatal arrowshot head trauma (AHT) as well as blunt force trauma (BFT) on cranial and postcranial remains^d^ and their timing during the peri = perimortem and post = post-mortem phases; chronology (phases of occupation); Radiocarbon data (^e^MAMS = Mannheim AMS facility at the Curt-Engelhorn-Centre for Achaeometry; References. More detailed information as well as a Bayesian modelling of the Phase I data is to be found in Materials and Methods, Supplementary Text S2 and Supplementary Fig. S4).

ID	Age (y)	Sex morph.	Sex genet.	HVS I^a.b^	Y-chrom.^b,c^	Violence pattern^d^	Phases	Radiocarbon data cal. BCE^e^	References (genetic data)
CET 1	5–6	n.d.	f	J1c3	(-)	BFT peri and post	Phase I	5311–5218 (MAMS 16159)	^a,b^Haak *et al*. 2015 ^a^Szécsényi-Nagy *et al*. 2017
CET 2	> 20	m?	m	J1c1b	I2a1a	post	Phase II	3933–3709 (MAMS 16160)	^a^Szécsényi-Nagy *et al*. 2017 ^c^Villalba-Mouco *et al*. 2019
CET 3	30–50	m	m	pre-T2c1d2	R1b1	BFT peri and post	Phase I	5294–5066 (MAMS 16161)	^a,b^Haak *et al*. 2015 ^a^Szécsényi-Nagy *et al*. 2017
CET 4	3.5–4.5	n.d.	m	K1a2a	F*	BFT peri and post	Phase I	5294–5068 (MAMS 16162)	^a,b^Haak *et al*. 2015 ^a^Szécsényi-Nagy *et al*. 2017
CET 5	30–50	m	m	N1a1a1	I2a1b1	BFT peri and post	Phase I	5310–5080 (MAMS 16164)	^a,b^Haak *et al*. 2015 ^a^Szécsényi-Nagy *et al*. 2017
CET 6	25–40	m?	m	U3a1	I2a1a	post	Phase II	3946–3767 (MAMS 16165)	^a^Szécsényi-Nagy *et al*. 2017 ^c^Villalba-Mouco *et al*. 2019
CET 7	3–4	n.d.	f	V	(-)	BFT peri and post	Phase I	5303–5075 (MAMS 16166)	^a,b^Haak *et al*. 2015 ^a^Szécsényi-Nagy *et al*. 2017
CET 8	6–8	n.d.	n.d.	H1	n.d.	post	Phase III	3350–3101 (MAMS 16167)	^a^Szécsényi-Nagy *et al*. 2017
CET 9	> 30	n.d.	n.d.	n.d.	n.d.	post	Phase III	assigned stratigraphically	
CET 10	50–70	f	n.d.	K	n.d.	AHT and BFT peri and post	Phase I	5310–5078 (MAMS 16168)	^a^Szécsényi-Nagy *et al*. 2017
CET 11	6–7	f?	n.d.	n.d.	n.d.	BFT peri and post	Phase I	5312–5219 (MAMS 16163)	
CET 12	30–50	m	n.d.	T2	n.d.	BFT peri and post	Phase I	5218–5034 (MAMS 40100)	^a^Szécsényi-Nagy *et al*. 2017
CET 13	> 20	n.d.	n.d	n.d.	n.d.	AHT and BFT peri and post	Phase I	assigned stratigraphically	
CET-F22580	> 20	n.d.	n.d.	n.d.	n.d.	AHT peri	Phase I	assigned stratigraphically	
CET-F22567	> 20	n.d.	n.d.	n.d.	n.d.	AHT peri	Phase I	assigned stratigraphically

**Table 2:** Results of the Bayesian modelling (Phase analysis) for the radiocarbon dates associated with the human bones of the early (Phase I) occupation of Els Trocs cave. Calibration and modelled curve Intcal 13; programme OxCal v.4.3.2^66,67^.

TROCS I - PHASE BC Amodel = 108.7/Aoverall = 108						
	14 C yr BP	Unmodelled cal. yr BCE		Modelled cal. yr BCE		A (individual agreement indices)
		1σ (68.2%)	2σ (95.4%)	1σ (68.2%)	2σ (95.4%)	
End of End				5225–5158	5284–5067	
Period of End				0–3	0–129	
Start of End				5277–5190	5290–5132	
End Trocs I				5226–5177	5285–5108	
CET 12	6175 ± 31	5207–5071	5218–5034	5282–5192	5291–5147	59.4
CET 3	6217 ± 25	5226–5076	5294–5066	5284–5204	5296–5148	117.5
CET 4	6218 ± 24	5282–5077	5294–5068	5283–5205	5296–5141	122.3
CET 7	6234 ± 28	5299–5084	5303–5075	5283–5208	5296–5203	136.1
CET 5	6249 ± 25	5295–5215	5310–5080	5281–5212	5290–5209	110.7
CET 10 (X)	6249 ± 28	5296–5215	5310–5078	5281–5211	5292–5208	114
CET 1	6280 ± 25	5302–5229	5311–5218	5257–5214	5293–5212	92.8
CET 11	6285 ± 25	5304–5229	5312–5219	5257–5214	5294–5212	91.5
End of Start				5289–5215	5317–5208	
Period of Start				0–28	0–103	
Start of Start				5308–5219	5358–5214	
Start Trocs I				5299–5218	5326–5214	

**Figure 2:**
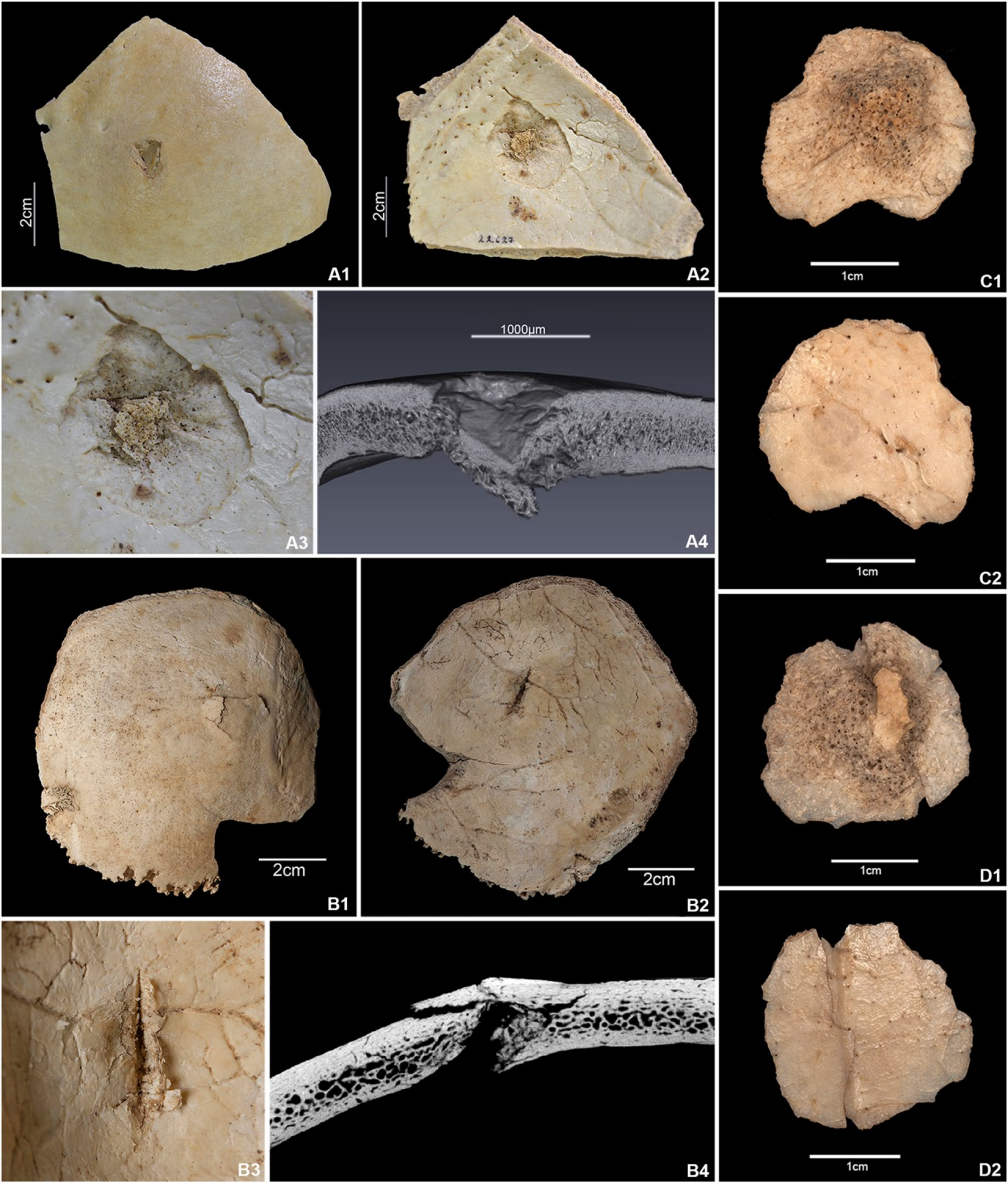
Four cases of fatal arrow-shot injuries from Els Trocs cave site. (**A1**) Fragment of the left parietal bone of individual CET-10, external view. The triangular lesion was produced by localized blunt force non-penetrating trauma (arrow shot) impacting the surface at high speed. (**A2**) Internal aspect of A1. The impact reached the internal lamina and lead to the splintering of a fragment, producing a funnel-shaped defect crater. (**A3**) Detail of the ca. 24 mm long funnel-shaped defect; the detached fragment is missing. (**A4**) Micro-CT image of the lesion in the parietal bone of CET-10 with the section plane running through the lesion. It shows the external depression and internal protrusion of bone fragments resulting from the impact of the arrow. (**B1**) Fragment of the right parietal bone of individual CET-13, external view. Visible are the fracture lines of the external protrusion with splintering. (**B2**) Internal aspect of B1. The slit-like cut at the internal point of impact causing the external protrusion. (**B3**) Detail of the slit-shaped defect due to blunt force trauma. The flint arrowhead penetrated the left side of the skull, traversed the brain and obliquely lodged in the parietal bone opposite the point of penetration. (**B4**) Micro-CT image of the area of the flint arrow defect of CET-13, showing a continuous, deep defect with protrusion of the external lamina. (**C**) Example 1 of an isolated roundish fragment (dislodged funnel-shaped cranial bone fragment, ID 22580) dislocated by the impact of an arrow shot from a parietal bone in external (**C1**) and internal (**C2**) view. (**D**) Example 2 of a defect funnel fragment (dislodged funnel-shaped cranial bone fragment) dislocated from a parietal bone (ID 22567) in external (**D1**) and internal (**D2**) view. Both fragments closely resemble the defect crater in CET-10 (A2 and 3), tapering from the internal lamina to the diploe, but only one fragment (D1) contains portions of the external lamina. ID number = isolated bone; CET-number = skull (photos: T. Schuerch; micro-CT images: G. Schulz using a Phoenix nanotome®m).

**Figure 3:**
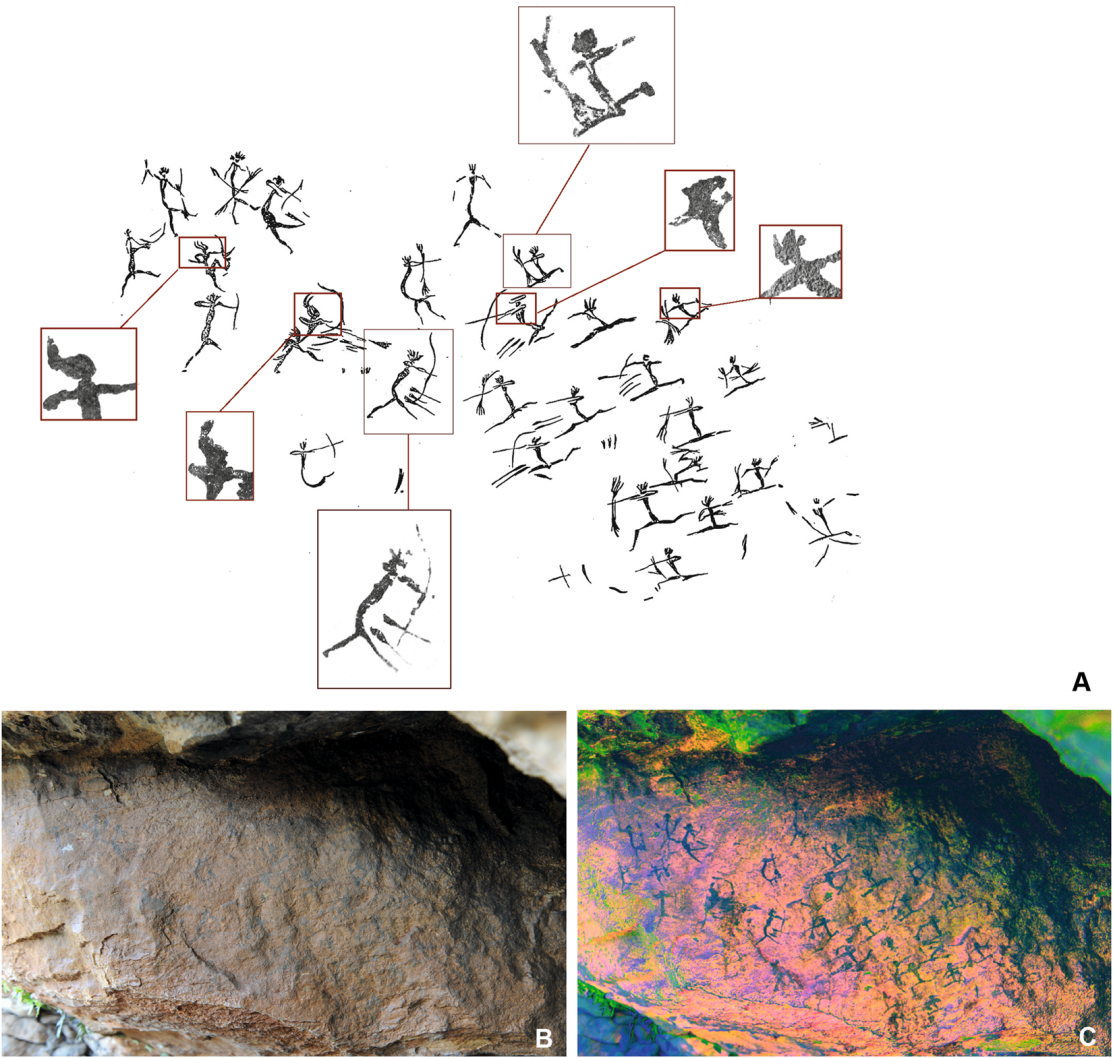
Battle scene from the Les Dogues rock shelter (Ares del Maestre, Castellón, Spain). (**A**) Digital tracing made by EL-M based on Porcar, 1953 (Supplementary Text S3). Up to 29 warriors organised in two opposite camps are depicted. The attack, carried out by the group on right, might be headed or controlled by the unarmed man at the top center of the scene. Both personal ornaments and anatomical proportions serve to differentiate the opposing groups and certain members of each camp. (**B**) Image of the panel of Les Dogues. (**C**) D-Stretch for Image J adjustment of Les Dogues rock art panel (Illustration: E. López Montalvo).

With regard to their chronological and osteological context, the traces of violence on the human remains from the Early Neolithic suggest a singular episode of conflict to which these individuals fell victim. The adults display consistent arrow-shot injuries to the skull but not to the perpendicular skeleton (Fig. 2, Supplementary Fig. S1). The children and adults furthermore show traces of similar blunt violence to the skull and entire skeleton (Supplementary Fig. S2, Text S3). The use of projectile weapons such as bows and arrows in conflict situations is evidenced not only by bows from the nearby contemporary wetland settlement of La Draga^10^, but also by rock paintings from this period depicting various types of violent acts (Fig. 3, Supplementary Fig. S3). Such, including battle scenes between hostile groups, exist in rock shelters on the Iberian Peninsula^11^. In addition to the direct, unambiguous traces of violence, this indirect evidence supports the assumption that the individuals in Els Trocs became the victims of a massacre (Supplementary Text S4).

The extraordinary significance of the violent conflict presented here lies in the early evidence of intentional violence in the Neolithic period. The existing sources date the first events of collective violence, to which entire settlement communities fell victim, chronologically to the final phase of the first farming culture (LBK) in Central Europe at the end of the 6^th^ and beginning of the 5^th^ millenium BCE, a phase of upheaval and change^12–16^. The violent events in Els Trocs are without parallel either in Spain or in the rest of Europe at that time. Another unusual feature is the remote geographical location of the site, away from the early Neolithic migration routes on the Iberian Peninsula, which are located inshore or along the Ebro valley, respectively^17^. From a theoretical and analytical point of view, the complex violent findings on the skeletal remains of Els Trocs raise two fundamental questions: on the one hand about the assailants, on the other hand about the motive for a seemingly uninhibited excess of violence visited on a group of older adults and children.

The question of the perpetrators cannot be answered directly. Archaeoforensically, they left hardly any evidence. Based on the overall context, however, alternative scenarios can be described and their probability evaluated. Population genetic analyses characterise the victims from Els Trocs as early Neolithic migrants, members of the communities that established farming and animal husbandry on the Iberian Peninsula^17,18^ (Supplementary Text S5). Judging from the strontium and oxygen isotope data (unpublished data) and the archaeological context, it cannot be decided whether the victims were among the first generation of migrants. While most Neolithic migrants reached Western Europe from the Fertile Crescent via a Mediterranean route, it cannot be ruled out that the phase I individuals from Els Trocs came from the north via the Rhone Valley. The grounds for this assumption are the genetic profile set of the group. One adult male from Els Trocs (CET 5) exhibits mtDNA haplogroup N1a1a1, which was common in early Neolithic Central Europe but previously unknown in Spain^19^. He represents the oldest early Neolithic individual on the Iberian Peninsula matching a Central European Neolithic mtDNA haplogroup. Only recently has new evidence been published of other N1a individuals along the hypothetical migration route from Central to Southwest Europe through France^20,21^.

Why, however, did this group from Els Trocs suffer a different fate than other early migrant groups in Spain, who relatively quickly formed mixed populations with the indigenous communities?^18^. Was their doom possibly dictated by the isolated geographical location, slightly off the Iberian Neolithic migration mainstream? The archaeological context, zooarchaeological and archaeobotanical data as well as the demographic composition of the group of victims suggest that these might represent the older members and children of a larger Neolithic community who, separated from the main group, spent the summer months in the Pyrenees in the course of seasonal transhumance, i.e. livestock migration^22,23^.

Two hypotheses can be formulated for the isolation of the perpetrator group and their motives. If the causes were territorial in nature, for example, then the perpetrators could have been indigenous hunter-gatherers who saw the Neolithic group encroaching upon their foraging territories, and who may have brutally enforced their claims. Alternatively, it may have been an altercation between two Neolithic groups in which disputes over territorial rights escalated. This reasoning considers as a motive that the terrain on which the violent event took place is a plateau offering manifold resources. The other variant for interpreting the conflict focuses on general patterns of behaviour. Common causes of disputes between local groups, regardless of time and place, origin or ethnicity, are territorial disputes, raids for acquisition of possessions (e.g. livestock, women, harvest), and conflicts over scarce resources (land, water, game). In contrast to present day interracial or inter-ethnic conflicts (“interracial clash”), however, the systematic extermination of entire communities is rather rare in the event of the recurrent use of violence between neighbouring groups, who often know each other well and may share socio-cultural roots^3,24^ (Supplementary Text S6).

The dramatic composition of the events in Els Trocs may never be fully clarified in detail. The extent to which violence was exerted in the remoteness of the Pyrenees, however, reveals an extraordinarily high potential for aggression on the part of the attackers, a phenomenon as manifested in forensics as “overkill” or “killing frenzy”^25^. What is clear is that two rivalling groups fatally collided. These may either have been a local group still living traditionally as foragers, who would decide to oppose the migrants - representing the invaders - decisively and with full force (Supplementary Text S7). Or it may have been another, either foreign or locally competing Neolithic group disputing the victims from Els Trocs their summer pastures. From the perspective of a criminal profiler, a coincidental encounter which escalated seems scarcely imaginable: the procedure appears systematically planned and executed, the motive for the act serious. So, with regard to both the local setting and the archaeological evidence, these several possibilities underlying the tragic events at Els Trocs must remain unresolved.

Violent conflicts between neighbouring groups (states), between different ethnic groups within multi-ethnic societies and between ethnic minorities and majority populations are often based on power politics exploiting the fear of difference in regard to people’s appearance, language, religion, ideology, way of life and cultural or ethnic affiliation. Countless examples of conflicts of the present and the past have shaken our view of overcoming a fundamental disposition to violence in human societies by civilisation^26,27^. If violence serves to defend oneself or to protect the community, it is socially tolerated^28^. However, if it aims at exploitation and power against third parties or against the welfare and life of peaceful people, it has a negative connotation. In its structural form, violence helps to exercise power and is thus undoubtedly a product of our cultural evolution^29^. In a world that is repeatedly shaken by nepotism, treaties and laws always seem to make peace only temporarily. Epochal crimes against humanity were never prevented by legislation or international law (Supplementary Text S8).

What conclusions may we draw, with all due caution, with regard to the events in early Iberian Neolithic in comparison to the present - a present marked by numerous ethnic and other inter-group conflicts that shake the world? Phylogenetic and neurocriminological studies provide some evidence that violence and crime have biological roots^7,30^. However, no human being is born a murderer and social processes control our behaviour profoundly. With regard to life in groups, evolutionary biology argues that humans have been both dependent on and threatened by other individuals since the beginnings. Therefore, mechanisms should exist which, on the one hand, cause a strong bond to one’s own group, close and closest kin, and on the other hand, fear and rejection of foreign individuals. The concept of ethnic nepotism serves as a causal explanation for this dual behavioural tendency^31^ (Supplementary Text S9). Vanhanen^32^ sees the roots of ethnic conflicts in this ethnic nepotism. Therefore, conflicts would occur more often in heterogeneous multi-ethnic societies than in homogeneous societies, where people are more closely related and connected^33^. Hypotheses on the origin of nepotism focus on sociobiological theories such as inclusive fitness, kin selection and genetic similarity^34^. Since an undeniable tendency towards ethnic nepotism seems deeply rooted in human nature, it can promote ethnocentrism, nationalism, racism and xenophobia in political or social crisis situations, in the worst case even culminating in genocide^35^.

So is there a “virus of violence” in every society? Is it one of the cultural universals of the present day that one’s own origins and membership of a particular religious community or cultural group are in many cases valued more highly than the achievements of civilisation in a modern open society, a society in which people of different nationalities and languages live together peacefully in freedom of religion and comfortable interactions between men and women? Does the vision of multicultural societies remain a fiction despite migration, intermixing and globalisation? Do different traditions, lifestyles and values at best allow parallel societies? (Supplementary Text S10). Postcolonial, the idea of a peaceful prehistory and non-violent tribal societies was very popular^36^. In the meantime, the theory that violence within and between groups is a modern phenomenon has been refuted. On the contrary, it manifests behaviour present in human history since time immemorial^37^.

Els Trocs probably documents an early escalation of inter-group violence between people of conceivably different origins and worldviews, between natives and migrants or between economic or social rivals. The conflict conveys the impression of a xenophobic action; the type of aggression suggests a clash between enemy groups. Such conflicts also occur among some social animal species: “Chimpanzees, like humans, divide the world into ‘us’ versus ‘them’” comments Russell^38^ (p. 111) on the behaviour of our primate cousins. In humans, therefore, violence is to be understood in the sense of a natural phenomenon. The “uniqueness of humans to do the things we do - albeit for good and for ill” can therefore limit violence, but never completely overcome it^39^ (p. 555).

The “civilised” present is still characterised by discrimination, intolerance and violence, by armed conflicts between neighbouring countries and aggression against national minorities within “mono-ethnic” states. Racism, nationalism and nepotism form their breeding ground and at best are regulated through national cultural control and international consensus. Promoting education, prosperity and the reduction of inequality may be the means to ensure a fairer world^40^. Our common origin lay in Africa, where the migration and spread of *Homo sapiens* began throughout the globe. In the meantime, globalisation and worldwide migration is once again making neighbours of people from afar^41^. On closer inspection, it seems that it is less our nature than our cultural diversity that impedes universal peaceful coexistence. A sustainable future will therefore only be achieved through mutual respect, tolerance and openness to multi-ethnic societies as well as the elimination of barriers between cultures and religions by on-going dialogue (Supplementary Text S11).

## Materials and Methods

The analyses carried out thus far (osteoanthropology, archaeogenetics, isotope analysis, radiometric dating) were exclusively performed on cranial remains that can be associated with one of the 13 individuals recorded and listed in Table 1, which also make up the current minimum number of individuals (MNI).

### Osteologic data

Based on individualized skull fragments, some with the associated maxilla and teeth, a cranial MNI^42^ of 13 was established (Table 1). These 13 individuals scatter over three occupation phases (see chapter Radiocarbon data below). In this paper, we only consider the 9 individuals from first, i.e. oldest phase of occupation of Els Trocs. Determinations of sex and age at death are based upon standard osteological methods. The biological age estimation of subadults is based on their tooth development^43^. For age estimation of the adult cranial individuals, molar abrasion^44^ and cranial suture synostosis^45^ were used. Adult sex is based in four cases on specific morphometric features of the skull^46–48^, which in two cases was confirmed by genomic data (see below). For skull CET 13, sex is as yet undetermined. Sex of 3 of 4 children was exclusively established by genomic data (see below).

At first appearance the age structure of the 9 individuals from phase 1 revealed a nearly balanced ratio of 4 subadults and 5 adults (Table 1). However, the four subadults are all between 3 and 7 years of age, whereas the 5 adults are invariably older individuals. Such a demographic structure where older children, adolescents and young adults are missing does not correspond to a natural age- and sex-balanced social community^49^. Taking into account the archaeological context, they were most likely the youngest and oldest members of a valley-farming group that practiced seasonal transhumance in the mountains.

### Identification of peri- and post-mortem trauma in the skeletal remains from Els Trocs

The basis for the palaeopathologic and archaeoforensic assessment of trauma in cranial and postcranial skeletal elements is again the group of 9 early Neolithic individuals from phase 1 (Table 1). For diagnosis and interpretation of the fatal injuries in adults and children, standard works in osteology, palaeopathology and forensic medicine as well as comparative literature were used^2,48,50–55^. After a violent death, traces of injury may be left behind in the skeleton. From an archaeoforensic point of view, all fractures and defects on bones are initially considered as signs of injury until proven otherwise. Blunt and sharp violence as well as the presence of projectiles combined with corresponding injuries represent clear evidence of interpersonal violence. However, determining the time when injuries occurred is more difficult^56,57^. When establishing the cause of death, only injuries which occurred around the time of death are relevant, i.e. perimortem trauma. Perimortem trauma is clearly distinguished from injuries sustained during life, i.e. injuries which were survived. The majority of bone changes usually occurred after death, i.e. post-mortem. Post-mortem taphonomic changes are caused by intentional and non-intentional impact by humans or animals while bone is exposed or in the ground (weathering, fragmentation, trampling) or during recovery. Distinction of intravitam, perimortem and taphonomic processes is essential for assessing the time when specific changes arose. However, demarcation of the perimortem and post-mortem phases is highly variable and dependent on the specific context.

The most important criterion for assessing post-mortem change is the color of fractured surfaces, which is usually significantly lighter than the rest of the bone surface. Further criteria are lack of or differences in decomposition at the fracture surfaces in comparison with the remaining bone tissue. Fracture edges appear more blunt edged and faceted with increasing time since deposition. Post-mortem lesions on the cranium have irregular to sharp curved edges^57^. Survived bone trauma shows signs of bone healing and remodelling whereas perimortem or post-mortem bone changes clearly do not^48,58^. Traces of injury that neither show evidence of healing nor are of post-mortem origin are classified as perimortem and thus may have occurred in connection with the death. Perimortem lesions are usually of the same color as the rest of the bone surface, and exhibit similar signs of taphonomic change^57^.

Direct fractures of the skull occur as hole, terrace or globe fractures (so-called bending fractures). In the case of a perforated hole, a piece of bone appears to have been punched out of the skull. If perforation is incomplete and a bone segment is merely driven into the interior, this constitutes a terrace break. If the impact surface is larger, circular fracture lines may form around the area of impact and linear lines run out radially (globe fracture). Fractured edges can be right-angled or obtuse in profile^48^. Partly, such blunt force trauma may generate additional hairline fractures ending discreetly in the middle of a skull bone^50,51^. In contrast to bending fractures caused by direct violence, indirect burst fractures caused by transmitted violence are more often found at the base of the skull.

Postcranial bone injuries show different characteristics than those observed on the skull^59^. Perimortem blunt force trauma often causes either classic “butterfly” fractures on long bones, i.e. a bend fracture with a flexion wedge or typical spiral fractures in torsional motion. Other fracture types of the perpendicualar skeleton such as transverse, oblique or comminuted fractures are more difficult to assess as they may be closely imitated by post-mortem lesions. The edges of perimortem fractures on the postcranium are frequently slightly curved to spiral in shape, as well as being sharply demarcated and exhibiting a smooth surface. Occasionally small compact fragments are broken out^60^. In contrast, post-mortem fractures on the postcranium show a jagged or linear, irregular, slightly granular and differently colored ruptured edge surface^53^. These fractures are mostly transverse.

All excavated skeletal elements from Els Trocs were examined macroscopically and with a 30x magnifying glass, some fragments in more detail with a 80x–100x binocular microscope. Among the 9 individuals from phase I, 2 of the 5 adults were diagnosed with lethal arrow wounds. In addition, all 9 individuals had suffered blunt force trauma to the skull and postcranial skeleton. All of these lesions represent intentional perimortem violence, with the most important diagnostic criteria being the localization, type and shape of the lesions, the shape of the fracture edges and the typical funnelling in cranial defects^2,54,61^. Further taphonomic post-mortem changes are observable in the human skeletal material, which occurred during the period of deposition and are unconnected with the violent event, which the individuals fell victim to (Table 1, Supplementary Text S2).

### Ancient DNA analyses

The skeletal remains from the cave at Els Trocs were part of a population genetic study which focussed on the settlement history of the Iberian Peninsula between the Neolithic and Early Bronze Age. Mitochondrial DNA profiles of ten individuals (CET 1 to 8, CET 10, CET 12; see Table 1) from Els Trocs were previously processed and published by our research team^17^. Sample preparation, DNA processing and quality control as well as population genetic analyses followed standard protcolls^17,62^. This current study also includes seven individuals (CET 1 to 7; see Table 1) analysed in cooperation with the Max-Planck Institute for the Science of Human History in Jena (Germany) and the Department of Genetics, Harvard Medical School, Boston (US). Samples were first screened for estimation the preservation of the genomic DNA content, then genome-wide distributed 1240k SNP were captured and sequenced^18,19,63^. Genetic sex was determined based on the ratio of sequence reads mapped to the X and Y chromosomes. Data mentioned in this paper are the result of the analysis listed in Table 1.

### Radiocarbon data

Existing radiocarbon dates document three Neolithic phases of use for the Els Trocs cave between 5300 and 3300 cal. BCE. There are radiocarbon dates for 11 of the 13 individuals constituting the present MNI at Els Trocs (see Table 1). No radiocarbon dates are as yet available for CET 9 (phase III) and CET 13 (phase I) as well as for the two dislodged funnel-shaped cranial bone fragments CET-F22580 and CET-F22567 (Fig. 2C,D). As conjoining pieces for these isolated fragments may come to light in future excavations, sampling was for now postponed. All radiocarbon dates were measured at the Mannheim AMS lab (MAMS) at the Curt-Engelhorn-Centre for Achaeometry, Germany, based on the compact MICADAS-type spectrometer (Mini Carbon Dating System)^64,65^. The results listed in Table 1 are calibrated at the 2σ confidence level (95.45 probability) using OxCal 4.3.2^66^ (https://c14.arch.ox.ac.uk); IntCal13 atmospheric curve^67^. Both the standard calibration and Bayesian modelling confirm that phase I can be attributed to a single event (Table 2, Supplementary Fig. S4).

## Supplementary information

Supplementary Information.
