# Supplementary material for: A massacre of early Neolithic farmers in the high Pyrenees at Els Trocs, Spain
Source: Sci Rep. 2020 Feb 7;10:2131. doi: 10.1038/s41598-020-58483-9 (PMC7005801; doi:10.1038/s41598-020-58483-9)
Supplement: Supplementary file 1 — Supplementary Information. [file 41598_2020_58483_MOESM1_ESM.pdf]

Supplementary Information

A massacre of early Neolithic farmers in the high Pyrenees at Els Trocs, Spain

Kurt W. Alt, Cristina Tejedor Rodríguez, Nicole Nicklisch, David Roth, Anna Szécsényi Nagy, Corina Knipper, Susanne Lindauer, Petra Held, Íñigo García Martínez de Lagrán, Georg Schulz, Thomas Schuerch, Florian Thieringer, Philipp Brantner, Guido Brandt, Nicole Israel, Héctor Arcusa Magallón, Christian Meyer, Balazs G. Mende, Frieder Enzmann, Veit Dresely, Frank Ramsthaler, José Ignacio Royo Guillén, Eva Scheurer, Esther López Montalvo, Rafael Garrido Pena, Sandra L. Pichler, Manuel A. Rojo Guerra

Corresponding author: Kurt W. Alt; kurt.alt@dp-uni.ac.at

This supplement comprises

Supplementary Figures S1-S4

Supplementary Texts S1-S11

References for Supplementary Texts

Supplementary Figure S1

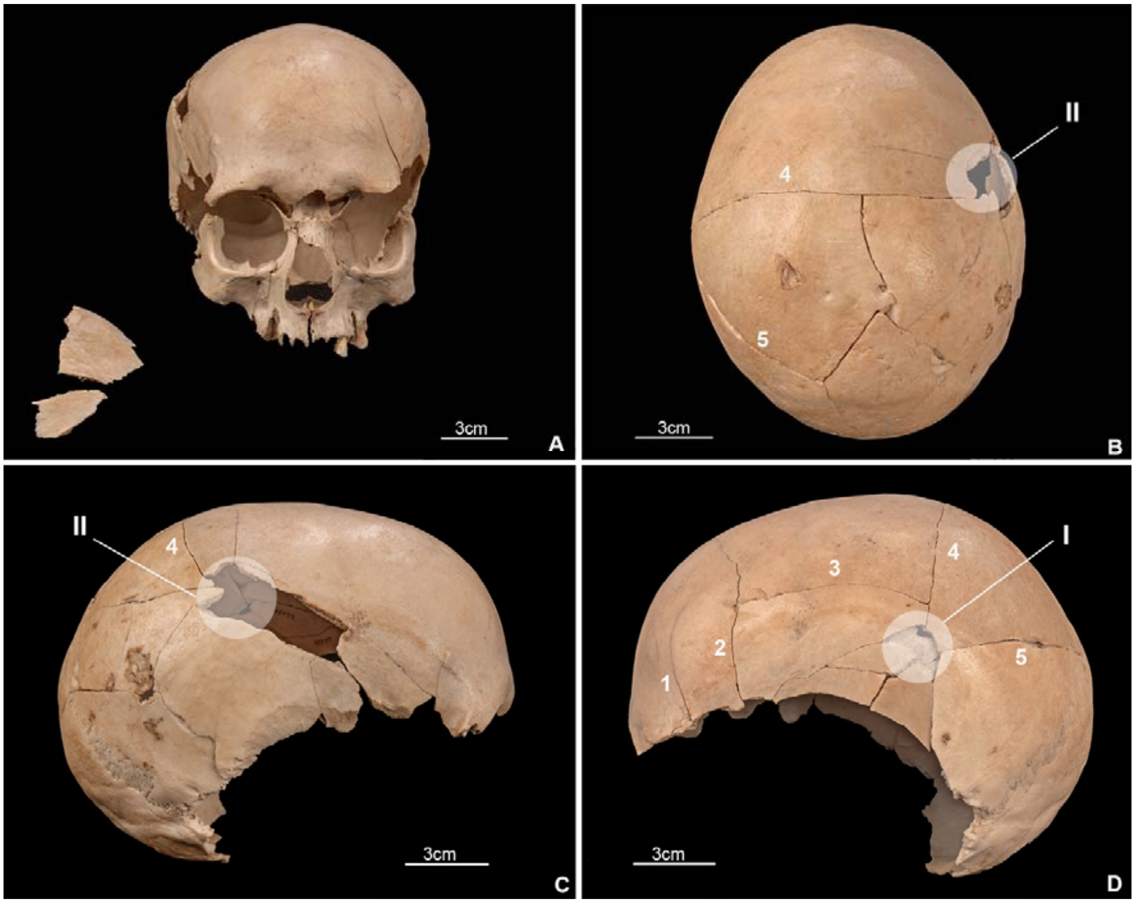

Figure S1. Individual CET 10 exhibits traces of multiple blunt force trauma to the face, both the right and left side of the skull as well as to the top of the head. (A) The face shows so-called Le Fort II and III midfacial fractures, with the point of impact possibly at the missing bone fragment in the glabellar region above the nose. (B) The blunt force arrow impact defect on top of the skull is clearly visible in the left parietal bone, impacting from above and behind. Lesion II marks the impact of penetrating blunt force to the right side of the skull. (C) Defect II is the result of at least two perimortem blunt force blows. A narrow rectangular object created a terraced fracture that caused the upper lesion in the right parietal. Underneath, the second impact formed a wedge-shaped lesion at its posterior end. Fracture line 4 traverses the skull and links lesions I and II, both of which basically exhibit radiating and concentric fracture lines. The terminations of the fracture lines radiating from II (B) suggest that the injuries on the right were inflicted after those incurred on the left side. (D) At least two blunt force impacts are visible on the left side of the skull. The fissure lines running almost parallel up the frontal bone (1 and 2) are probably the result of a blunt force impact on the missing left sphenoid bone. As fracture line 3 terminates in line 2, the trauma causing lesion I occurred after the sphenoid impact (photos: T. Schuerch).

Supplementary Figure S2

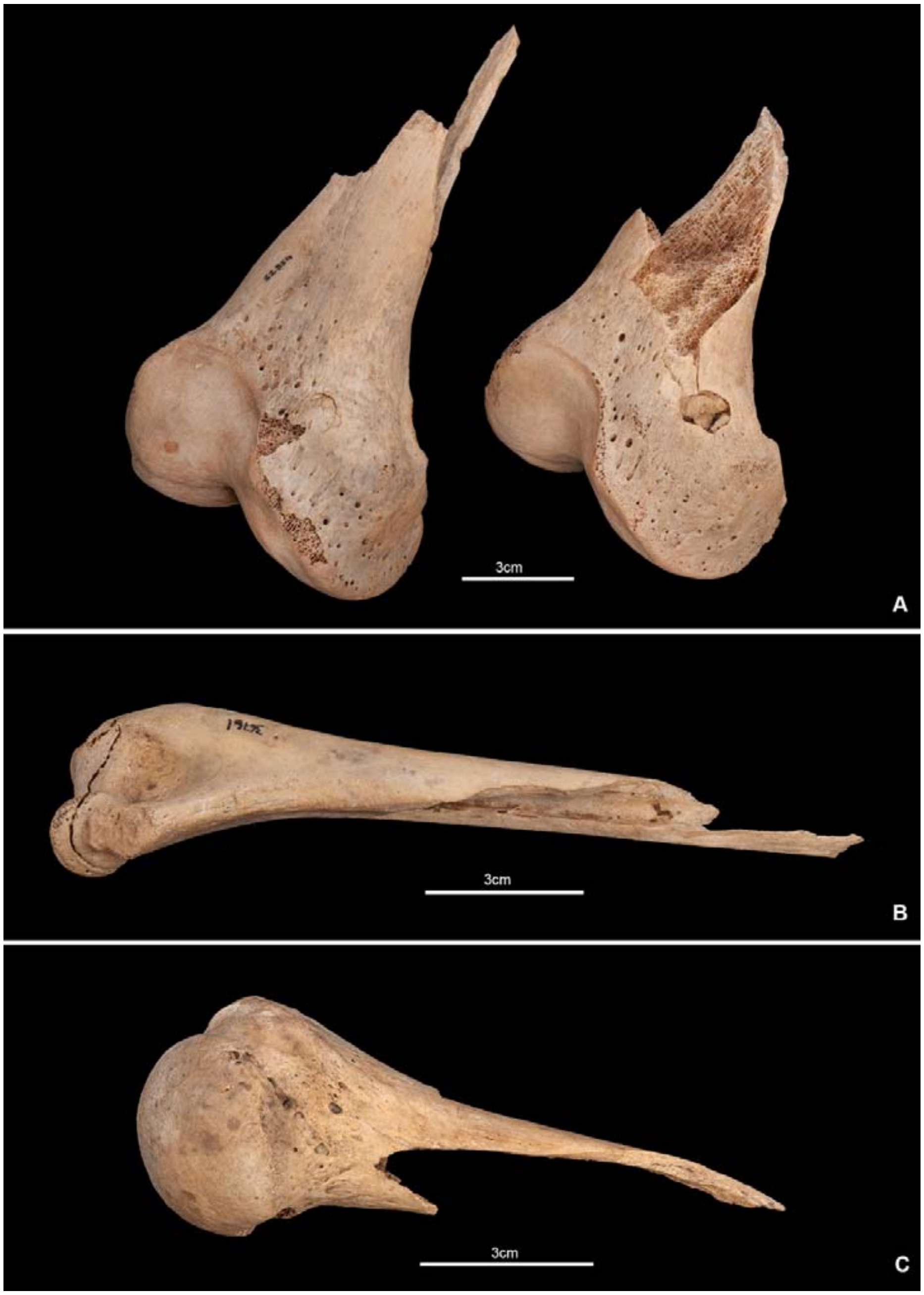

Figure S2. Typical patterns of violence on the postcranial skeleton of the victims in Els Trocs. (A) Frontolateral view of distal fragments of two right femoral bones (ID 22854 and ID 33575). Both bone shafts show perimortem wedge-shaped and spiral fractures. In addition, there are small, roundish perimortem impressions just above the knee joints inflicted by a pointed angular object. (B) Lateral view of a right humeral fragment (ID 36761) with a pointed perimortem fracture pattern and torsion of the fracture lines in a clockwise direction. (C) Proximal portion of a right humerus (ID 36808) with a typical spiral fracture just below the upper epiphysis (ID number= isolated bone; photos: T. Schuerch).

Supplementary Figure S3

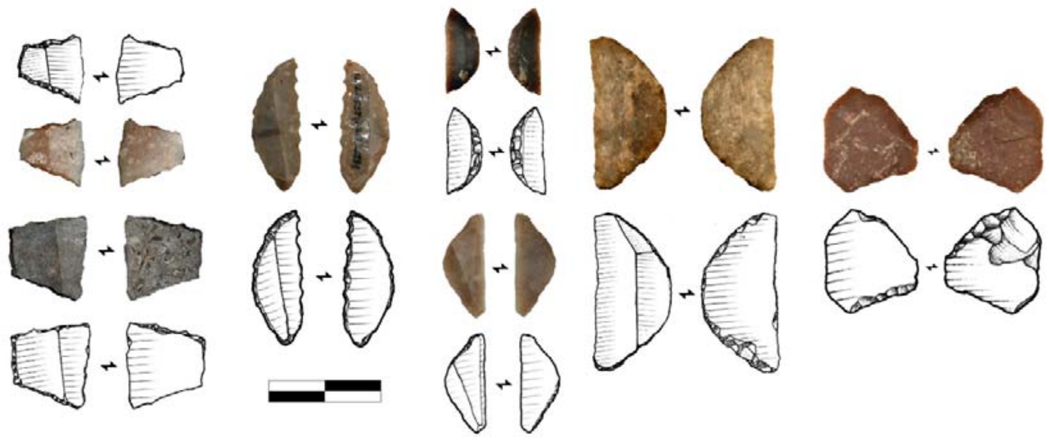

Figure S3. Flint inventory from Els Trocs. Set of flint geometric microliths from the cave of El Trocs. Use-wear analyses show that such geometric microliths were frequently used in projectile points and arrowheads during both the Mesolithic and Neolithic periods[1-3]. In the case of Els Trocs, distinct impact marks and fractures on most pieces confirm such a use[4]. Being typologically indistinct, the lithics from the cave may have originated either with the Neolithic group occupying the cave or the perpetrators responsible for their killing (photo: courtesy Niccolò Mazzucco).

Supplementary Figure S4

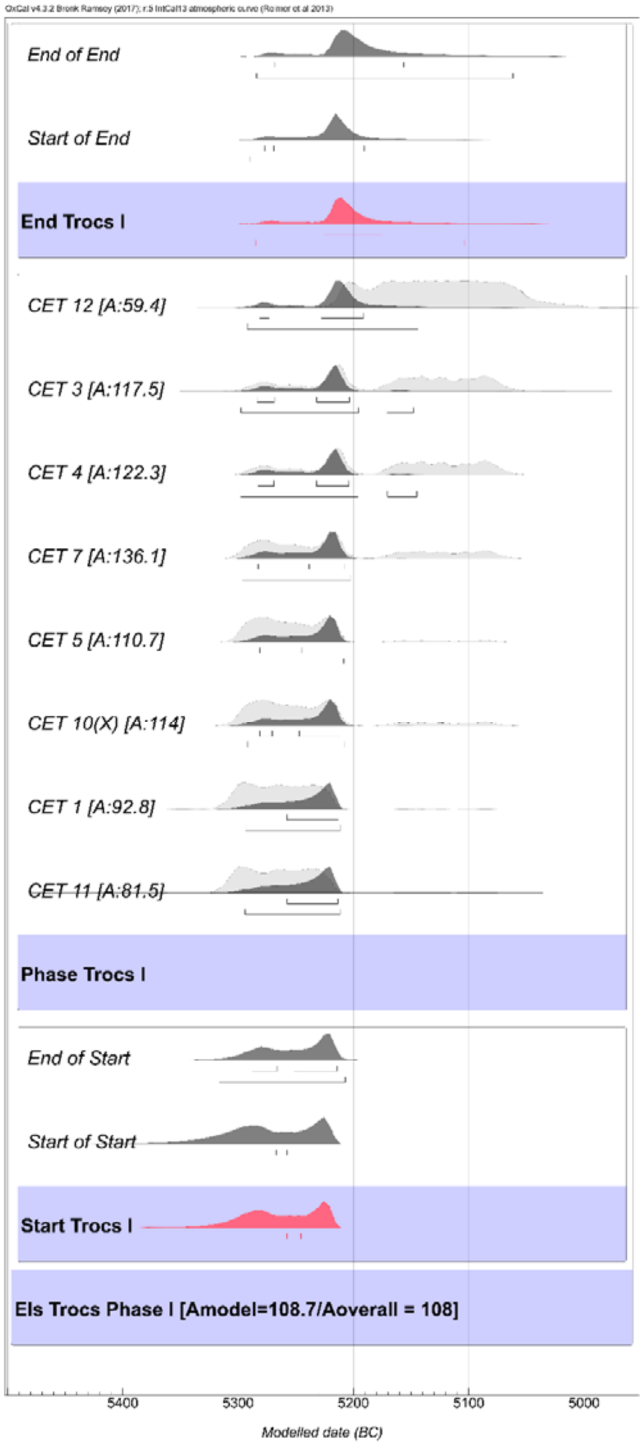

Figure S4. Bayesian modelling (Phase analysis) for the radiocarbon dates associated with the human bones of the early (Phase I) occupation of Els Trocs cave (ash grey curve=calibrated dates; dark grey curve=modelled dates; A=individual agreement indices). Also depicted are the boundary estimates for the Els Trocs I phase. Calibration and modelled curve Intcal 13; programme OxCal v.4.3.2.

List of Supplementary Texts

Text S1. Development of interpersonal and inter-group violence in the past
Text S2. Individual and archeometric data: Excavation history
Text S3. Archaeoforensic description of the victims' injury patterns
Text S4. What happened after the deadly assault?
Text S5. The Neolithization of Western Europe
Text S6. Violence as virulent factor in the society today
Text S7. Neolithic population admixture and the beginning of multi-ethnic societies
Text S8. Power politics and modern ethnic violence
Text S9. The origin and universality of ethnic nepotism from the perspective of behavioral biology
Text S10. Ethnic identity which promotes inequality between groups is a threat to any society
Text S11. Key factors for the world of tomorrow

Text S1. Development of interpersonal and inter-group violence in the past.
Norbert Elias[5] outlined the process of civilisation as a gradual development towards the disappearance of overt violence. This view is effectively disproved by recent violence statistics such as the Global Peace Index, which records the violent conflicts that are ongoing throughout the world (in 163 countries)[6]. In terms of human evolution, it seems justified to raise the fundamental question of whether *Homo sapiens* would have prevailed without the use of violence. In a hostile environment with numerous competitors vying for the same sources of food, violence is a phenomenon that exists as part of human nature and seems indispensable for the protection of the community. It was, after all, the killing of animals for the procurement of food that ensured the survival of the collective[7]. However, as seen in indigenous present-day communities, the killing of animals would probably also not have been taken lightly by prehistoric humans[8,9]. As a violent act hunting is generally embedded in a spiritual belief system where compensation must always be offered when something is taken from nature to secure one's subsistence.

Due to the limited number and differential preservation of fossils, the importance of interpersonal violence amongst early humans remains largely unknown to us and can, at best, be reconstructed by extracting information from isolated cases[10-12]. A recent study showed that Neanderthals and palaeolithic modern humans exhibit similar frequencies of cranial trauma[13]. In her commentary, Mirazón Lahr[14] did however caution that great care should be taken to differentiate interpersonal violence and accidental injury, which may produce similar lesions. Beyond sporadic observations for the Palaeolithic, evidence of violent interactions increases from the end of the Last Ice Age[15]. Early intergroup conflicts have been identified amongst hunter-gatherer communities from the Early Holocene in West Turkana, Kenya[16], and from the Mesolithic and Early Neolithic in Europe[17-19]. These violent clashes are usually seen in connection with sedentism and/or the appropriation of land by early farmers. The first

incidences of entire village communities being exterminated in central Europe appear to have occurred with the decline of the earliest farming culture (Linearbandkeramik=LBK) around 5000 BCE[20-24].

The question of whether these violent incidences in the LBK were, in fact, early armed conflicts or even war is a matter of debate and largely depends on the definition of the term »war«[25]. According to Otterbein[26], war is a social act that is sanctioned by society and directed at other groups; this quite clearly would not have existed in any organised form in the Early Neolithic. The armed conflicts from this period are better classified as feuds or raids and can, at best, be seen as the precursors to actual wars[27]. Studies of modern mobile forager bands show that the vast majority of such conflicts are individual homicides and feuds, with war playing hardly any role[28]. Confrontations between neighbouring groups in prehistoric periods are also much more likely to have been acts of revenge for earlier raids (cattle, women) than incidences of actual war[29,30]. The fact that the LBK victims of violence found at Asparn/Schletz (Austria), for instance, did not include any young women, can be seen as evidence of such a raid[24]. The demographic composition of the group of victims at Talheim (Germany) from the same period do not include equal segments of a living community either[31]. Some of the reasons cited for the extreme acts of violence that occurred at the end of the LBK include climatic changes, poor soil management (lack of nutrients) and overpopulation, leading to crop failures and periods of famine, and in some cases even to acts of cannibalism[32].

An increase in interpersonal and inter-group violence is detected as the Neolithic period progressed[30,33-36]. Certain researchers have taken the occurrence of collective violence in the 4th/3rd millennia BCE as marking the actual beginning of the age of war[37], though this statement refers to the availability of a more versatile arsenal of offensive weapons and a higher frequency of trauma identified in burials rather than to a real number of observed conflicts. The causes for the increase in violence in the Neolithic period are mainly seen in the context of radical social and economic changes that took place after the introduction of farming, including the appropriation of land, the accumulation of wealth, territorial boundaries, population growth, new social values and inequality. This new way of life led to the creation of hierarchies, mega-settlements and early states and, based on the intensification of mobility and migration, resulted in the formation of the first “multi-cultural” communities and an increasingly complex society. True acts of war involving organised collective violence, however, most definitely did not occur until the Metal Ages as a result of a further intensification in social hierarchies, the formation of political systems, the emergence of military castes and the development of specialised weapons systems[26,38,39]. A Bronze Age battlefield in the Tollense Valley in northeastern Germany with the remains of several hundred individuals is the earliest evidence of a professional violent conflict for which the label ‘war’ can be justified[40].

Thomas Hobbes (1588-1679), founder of enlightened absolutism, unlike any other scholar of his time, bemoaned in his writings the (natural) condition of humankind before the formation of social systems. In an exaggerated view of contemporary societal advances, his work *Leviathan* impressively describes human existence before the development of civil society as a “war of all against all” and goes on to state: “When all men were free and equal, no man was safe from any other. Life was short, fear knew no bounds. No law protected man from violation. No man trusted any other, and every man was forced to protect himself against his

neighbour”[41] (chapter 13). The core message of evolutionary psychologist Steven Pinker’s monumental work “*The better angels of our nature: why violence has declined*” was that: „A decrease in violence is probably the most important and least recognised development in the history of our species”[42] (p. 1027). This notion was widely accepted but is still much debated by scientists. The fact that Pinker believed the “process of civilisation” and the “humanitarian revolution” had led to a clear reduction of violence at the transition from the Middle Ages to the modern period is mainly attributed to the historical chroniclers whom he used as sources[43]. Whilst most researchers agree that the process of civilisation is accompanied by a decrease in violence, they nevertheless find fault in the fact that Pinker does not relate to the possibility of “fundamental processes of de-civilisation”, which some consider possible[5,44]. It is also noted that Pinker partially revized his own previously held convictions with regard to the constant aspects of human nature[45]. A high degree of optimism is required if one is to hope that the attributes ascribed to civilised human beings such as cooperation, affiliation, reconciliation, empathy, reason, morality, altruism and self-control will ultimately prevent the outbreak of a Third World War[42]. In this context, Falk & Hildebolt[46] point out that these qualities are as much the result of the biocultural evolution of humankind as is the development of biological, chemical and nuclear weapons, and that we will have to wait and see whether our civil responsibilities will be sufficient to prevent their proliferation and, ultimately, their use. Whilst the hypothesized use of untamed and unregulated spontaneous violence amongst humans in the prehistoric period was placed under a certain degree of cultural control when humankind became “civilised”, it was not overcome[47]. Therefore, the fact remains that we as human beings have always led and continue to lead the statistics amongst all mammals when it comes to violent acts against members of our own species[48,49]. The acquired civil status of human societies, our learnt social behaviours and our cultural advances, however, provide us with a diverse range of tools to curb the violent tendencies that are rooted in our evolutionary genetic makeup[50,51].

Text S2. Individual and archeometric data: Excavation history.

Table 1 shows the extent of the work that has been carried out to date on the skeletal material from Els Trocs cave (CET). Excavations commenced in 2009 and are still ongoing. Except for the near-complete skeleton of a child (CET-4), only disarticulated human bones have been found. And also unlike CET-4, the skeletal remains of the other individuals were widely dispersed throughout the cave, both horizontally and vertically. Furthermore, a large proportion of these bones were intentionally fragmented. Each new excavation campaign increases the prospect of finding more bone fragments and teeth that can be joined to the as yet incomplete skulls, jaws and long bones.

Text S3. Archaeoforensic description of the victims’ injury patterns.

Evidence of violence in all nine individuals from the earliest phase of use of Els Trocs predominantly consists of fatal arrow wounds to the head as well as non-specific blunt-force trauma to the cranial vaults and, in particular, the limbs. The arrow wounds can be divided into direct and indirect evidence. Direct evidence of an arrow wound was detected on the left parietal bone of individual CET-10 (case 1), appearing as a narrow, triangular bone defect on the *tabula externa* in consequence of a non-penetrating arrow shot (Fig. 2 A1-4). Another complex retained arrow shot wound was identified in individual CET-13 (case 2). Here, an

arrow had penetrated the left side of the skull with great force and had then lodged in the *tabula interna* on the opposite, right-hand side. The impacted projectile point was lost post mortem (Fig. 2 B1-4).

Case 1 refers to a female over 50 years of age (CET-10; Table 1, Supplemental Text S2), whose skull exhibits a depressed fracture near the *foramen parietale* and the completely closed sagittal suture. Shaped like an almost equilateral triangle, the fractured bone was pushed into the skull's interior (Fig. 2 A4). The lesion has a base length of 9 mm and an axis length of 11 mm and measures 3 mm at its deepest point, representing an incomplete depressed fracture with bevelled edges. The pattern of damage attests to a strictly localised impact by a triangular, semi-pointed or pointed object from a mostly orthogonal direction (Fig. 2 A1). The smaller spalls show that the force was of medium to high velocity.

The endocranial view of the skull shows that the lesion has affected the *lamina interna* with its typical funnel shaped widening (Fig. 2 A2, A3). The spalled-off endocranial splinters are significantly larger than the lesion on the outside of the skull where the original impact occurred (Fig. 2 A1). The funnel-shaped defect is vaguely rectangular with rounded corners and at its largest point measures 28 mm in length and 25 mm in width. The associated bone fragment was not recovered from the cave sediment. The skull of CET-10 was almost fully reconstructed from a total of 20 joining fragments (Supplementary Fig. S1). The majority of fragments were widely scattered in the same horizontal layer of the cave.

Case 2 is more difficult to assess from a traumatological point of view, because so far only one large fragment of the right parietal bone (CET-13; Table 1; Supplementary Text S2) has been identified. Significantly, the bone exhibits evidence of an endocranial arrow wound (Fig. 2 B2, B3). The damage pattern shows that the arrow penetrated the cranial cavity from the left side, with the projectile lodging inside the skull opposite the entry wound. The projectile must therefore have had a high kinetic energy and collision velocity. This is supported by the ectocranial protrusion (Fig. 2 B1-4) with external splintering (Fig. 2 B3) at the point of the internal impact, where the parietal bone is 4-5 mm thick. The somewhat asymmetric shape of the gash indicates that the arrow probably entered at a slight angle and perforated the thin temporal bone on the opposite side. Much like the brain tissue, the bone at the point of impact would have slowed down the projectile but little. The obviously thin flint arrowhead was at least 8 mm wide, suggesting that the projectile would have caused significant intracranial damage, with a large wound cavity and displacement of brain tissue also affecting central areas close to the brain stem. The injury can therefore be identified as the cause of death for this particular individual.

However, this is not all the evidence of intentional acts of violence that were identified on the skulls from Els Trocs. The numerous remains found in the cave sediment also included two small rounded bone fragments which represent endocranial sections of parietal bone (fragments CET-F22580 and CET-F22567). The spalls were relatively similar in shape and size and, from a forensic point of view, constitute dislodged funnel-shaped cranial bone fragments (Fig. 2 C1, C2, D1, D2). Such defects are the result of blunt force impact on the skull at high velocity[52]. The spalled-off fragments largely consist of parts of the *lamina interna* with traces of diploe and, in one case, the ectocranial lamina (Fig. 2 C1, D1). In relation to the ectocranial defect caused by the arrow at the point of impact, the endocranial parts on both of these

fragments are clearly extended or enlarged in the shape of a teardrop with a maximum length of 26 mm in one case (Fig. 2 C2) and 24 mm in the second (Fig. 2 D2). Both bone fragments closely match the intracranial defect on skull CET-10 in shape and size (Fig. 2 A2). However, a direct comparison excluded the possibility that either one of the isolated fragments came from either CET-10 or any of the other skulls recovered to date. They consequently constitute indirect evidence of further arrow-shot victims. Because of the similarities in shape and size in all three cases of direct or indirect evidence of arrow wounds, we may assume that they shared a similar genesis and were probably caused by similar weapons, i.e. bows and arrows. On the assumption that each of the victims was struck by one arrow, we can state that at least four adult individuals (Fig. 2 A-D) apparently died from arrow wounds as a result of a violent event in the high Pyrenees.

Besides the trauma caused by arrow shots, both skull and limb bones of the individuals analysed exhibit other, multiple traces of blunt force. The almost completely reconstructed skull of Individual CET-10, for instance, bears fractures to the face and to both sides of the skull, which were obviously caused by more than one blow (Supplementary Fig. S1-. The face shows the multiple lines of so-called Le Fort II and III midfacial fractures, with the point of impact possibly at the missing bone fragment in the glabellar region above the nose (Supplementary Fig. S1A). An arrow wound inflicted from above and behind and which was likely to be the initial lesion, described above as case 1, is visible in the view from above in Supplementary Fig. S1B. At least two perimortem blunt force blows impacted the right side of the skull (Supplementary Fig. S1C, II). A narrow rectangular object, creating a terraced fracture and driving the impacted bone fragment into the brain, caused the upper lesion in the right parietal. The second impact, possibly dealt with the same instrument, hit further down and to the front of the first, creating a wedge-shaped lesion at its posterior end. Fracture line 4 traverses the cranial vault and links the lesions on the right and left sides of the skull, both of which basically exhibit radiating and concentric fracture lines. The terminations of the fracture lines radiating from II (Supplementary Fig. S1B) suggest that the injuries on the right were inflicted after those incurred on the left side. The left side bears witness to further multiple external impacts (Supplementary Fig. S1D). Patterns that cause fracture lines ending at pre-existing fracture lines, attest to the sequence of injuries[53]. On the frontal bone, two fissured fracture lines are aligned sagittal, run almost parallel, and end in hairline fissures (Supplementary Fig. S1D). The exact point of impact on the left sphenoid bone is unclear since that part of the skull is missing. Just like Supplementary Fig. S1D impression I and the curving fracture line underneath, these are possibly the result of a hard fall, either following the arrow wound or the blow to the face. If that was the case, then the fatal injuries to the right side of the skull occurred when the woman was already on the ground. These multiple traumatic injuries inflicted on a victim already made helpless by the arrow shot suggest berserk overkill. Similar findings were also identified on the skulls of the other individuals from the cave's early phase of occupation.

Notably, most of the intentional long-bone fractures inflicted on these individuals exhibit the typical perimortem butterfly pattern, i.e. a pattern which results in a more or less wedge-shaped fracture, or are spiral fractures with characteristic slanting edges following the lines of torsion (Supplementary Fig. S2). Butterfly fractures usually occur at a low speed of impact[54] on stationary long bones (e.g., when the person is upright) and as a result of a roughly right-

angled blow. Axial loads, e.g., in falls or the violent rotation of long bones during a fight, on the other hand, produce spiral fractures.

Microlithic flints found in the Cueva Els Trocs have been archaeologically classified and analysed for traces of use[4]. The artefacts are retouched flint blades, which were traditionally glued onto arrow shafts to act as projectile points (Supplementary Fig. S3). Because both hunter-gatherer and Neolithic groups used such microliths, they cannot be specifically assigned to any of the two. Arrowhead technology remained essentially unchanged until the end of 4th millennium[55]. At that stage, bows and arrows began to play a more important role in violent clashes, due to enhanced aerodynamics and penetration power increasing their effectiveness. Evidence for the use of bows and arrows therefore becomes more prevalent from the Middle Neolithic onwards.

Scientific reports on understanding and interpreting violence in the past have significantly increased in number in recent years. Besides theoretical works on the origins of violence, these address methodological, traumatological, bioarchaeological and topics in forensic archaeology[56,57]. Knowledge on the technology of bows and arrows from prehistoric and historic periods has increased likewise[58]. Given the types of weapon available in the Early Neolithic, the wounds identified here were most likely caused by arrowheads made of flint, hardened wood or bone. The fact that in one case an arrowhead completely penetrated the skull is a clear indication of the high level of kinetic energy and velocity on impact of these projectiles (Fig. 2 B1-4). The contemporary wetland settlement of La Draga (Banyoles, Girona, Spain) with its excellent preservation of organic remains also yielded bows made of yew wood, which could have provided the necessary penetration[59-61]. Moreover, impressive rock art from the Iberian Peninsula firmly establish the use of bows and arrows at the time[62,63] (see Fig. 3).

Text S4. What happened after the deadly assault?

The numerous skull fragments that exhibited evidence of severe injuries inflicted by arrows were all from adult individuals dating from the cave's earliest phase of occupation. In view of their clustered radiocarbon dates and the identical patterns of violence, we may assume that they were the victims of a singular act of violence (see Table 2, Supplementary Fig. S4). Having died from their wounds or at least having become incapacitated, their injured or dead bodies must have quickly been removed from the site of the battle or raid and brought into the cave. From a taphonomic point of view, this is a reasonable assumption: on one hand because the bones exhibit hardly any gnaw marks or signs of exposure and on the other because even minute bones as well as joining fragments have been found dispersed throughout the cave sediment. This would not be the case if the bodies had been transferred to the cave after decomposition of the soft tissues. Other reasons for the suggested sequence of events are provided by projectile kinematics and the available space and light in the cave, which would make the use of bows and arrows inside it almost impossible.

Almost all bones from the individuals dating to the earliest Neolithic phase exhibit traces of both perimortem and postmortem violence. The typical perimortem butterfly and spiral fractures contradict the idea of a noticeable time lapse between the time of death and the initial blunt force exerted in shattering the bones of these individuals. The skeletal remains

from the younger phases of occupation, in contrast, exhibit only postmortem fractures quite unlike the perimortem lesions from phase I. This, again, confirms our interpretation that the group of individuals from the oldest phase of occupation suffered a single perimortem act of violence.

Text S5. The Neolithization of Western Europe.

Judging from the archaeological record, the expansion of early farmers from the Middle East to western and south-western Europe primarily occurred by means of direct maritime or coastal diffusion and migration. In the western Mediterranean region, the so-called Cardial Culture was the bearer of Early Neolithic development[55,64,65]. Technological, typological and cultural modifications suggest a gradual change in population due to Neolithic migrants between the Mesolithic and Neolithic periods, whilst acculturation of Mesolithic groups has also been considered as a possibility[64]. In recent years, the archaeological evidence has been underpinned by the extensive gathering of ancient genetic data, showing "that farming expansions across the Mediterranean to Spain and via the Danubian route to Hungary and Germany descended from a common stock"[66] (p. 208) However, this data also suggests that, following the introduction of farming, the population change on the Iberian Peninsula[67,68] was not as radical as that seen in central and south-eastern Europe[69,70]. There, admixture between larger groups of hunter-gatherers and early farmers occurred much faster than elsewhere in Europe. The Els Trocs cave site, which marks the chronological beginning of (seasonal) occupation of the Pyrenees, however, represents a notable exception; here we find evidence of violent opposition and conflict rather than a peaceful intermingling of indigenous and migrant groups or the amicable exploitation of landscapes based on a common economic approach.

Text S6. Violence as virulent factor in the society today.

Violence in its many guises is virulent in every modern-day society, be it within family units, marriages or partnerships, against children, foreigners, animals, in schools or in care. Violence between different ethnic groups is responsible for civil wars, riots in towns and cities as well as massive xenophobic conflicts in many states. Currently, social exclusion and discrimination of non-nationals is an oppressive social reality in many countries around the world. Over and above gender and class distinctions "ethnicity has obviously become one of the central dimensions of structure within the social order and an almost omnipresent framework of orientation for social action in multicultural societies"[71] (p. 7). Everyday conflicts appear to arise from differences in ethnicity and determine which type of conflict resolution is adopted and how the identities of those involved develop.

If these conflicts result in public acts of violence, it is usually in the form of riots, turmoil, rebellion and revolt, whereby active violations of state law are accepted as a natural component[72]. One of the main traits of such "deadly ethnic riots", according to Horowitz[73] is their irregular occurrence in space and time, their relatively spontaneous nature, the selection of the victims based on their membership of a particular group, the vehement rejection of the other group and the gratuitous mutilation of the victims. He makes a distinction between this form of "mass civilian intergroup violence in which victims are chosen by their group membership"[72] (p. 353) and other forms of violence such as genocide, lynch

law, violent protests, feuds and civil wars. The term ‘riot’ is used both for collective violence perpetrated by an ethnic group that perceives itself as disadvantaged and as a consequence turns against the state or a population majority, and for the violent assault of a majority against an ethnic minority. Janowitz[74] proposed a distinction between “commodity riots”, in which the focus of violence is the destruction of property and “communal riots”, in which one ethnic group attacks another (“interracial clash”).

Text S7. Neolithic population admixtures and the beginning of multi-ethnic societies.

The Neolithic transition after the end of the last Ice Age brought about the most radical economic and social change in the history of humankind. It involved gradually replacing the foraging lifestyle of hunters and gatherers (small group size, low population density) with a productive and sedentary economy and an increased population density. Besides changes in economy and material culture, this eminently significant process also led to crucial changes in all aspects of social life (e.g. ritual, symbolism, codex of values, community structure and organisation)[75]. Switching from a low to a high-carbohydrate diet supposedly soon triggered the so-called Neolithic Fertility Transition (NFT)[76]. A combination of new lifestyles and practices and increased sedentism led to a population explosion and brought about the Neolithic Demographic Transition (NDT), which was characterised by high birth and child mortality rates[77]. High population densities and pressure due to climatic events such as the 8.2 ka event[78] helped to set the scene for the rapid spread of a farming lifestyle from the Middle East to Europe. This signalled the evolution of multicultural communities like those found in almost all parts of the world today. Migration of foreign groups into Europe occurred in different ways in different regions, as suggested by archaeological and molecular genetic data on the Neolithic settlement and population history[66,69,79]. The molecular genetic profiles of the earliest groups of migrants arriving in central Europe via the corridor of the Carpathian Basin[80] differed quite distinctly from those of the Mesolithic foragers[81-83]. The hunter-gatherer groups of central Europe apparently largely retreated to northern Europe when the early farmers arrived. From a genetic perspective, there was hardly any admixture between indigenous groups and migrant populations in the Early Neolithic, and the same can be said for the Carpathian Basin. It was not until the 4th millennium BCE that there was a population-genetic reflux of hunter-gatherer lineages by way of the Funnel Beaker Cultures from southern Scandinavia into central Europe. The encounter of these two worlds that in some ways were ethnically and culturally quite diverse, was characterised by an increase in violent events throughout central Europe[84]. However, this general development notwithstanding, certain parallel communities of hunter-gatherers and farmers were also established in some places[85]. The introgression of the Funnel Beaker Culture in the 4th millennium BCE, appears to have marked the beginning of a heterogenous multi-ethnic society at least from a genetic point of view. This population dynamic culminated in the incorporation of Corded Ware Culture groups from the areas of the Eurasian steppe[66,69,79]. This became the foundation stone for today’s genetically homogenous European population, which originated from at least three ancestral sources: indigenous hunter-gatherer populations, early farmers from the Middle East and northern Eurasian steppe peoples[66,86,87]. Yet a different development took place on the Iberian Peninsula, where the Early Neolithic groups formed mixed populations with indigenous hunters and gatherers in the north-east of the territory much earlier than in central Europe, as demonstrated by molecular genetic data[67,68].

Text S8. Power politics and modern ethnic violence.
International law (Lat. *ius gentium*) is a supranational legal order of the community of states, which has governed the relationships between nations and international organisations since the 19th century (e.g. the International Committee of the Red Cross) on the basis of coequality and customary law. In cases of violent conflict, it distinguishes between wars and armed conflicts; the primary difference being that war is a planned operation that takes place between states and involves the mass deployment of weaponry and manpower, whilst an armed conflict comes about quite by chance and involves a violent confrontation without a central organisational structure. The law of nations was born in 1864 in Geneva. The First Geneva Convention was a humanitarian reaction to the atrocities of war and demanded assistance for wounded soldiers regardless of nationality. The modern extended version of international law is based on the Charter of the United Nations (Article 2 Figure 4) and its basic ban on the use of physical force. The legal order of international law, which consists of basic principles and rules, is constantly updated[88,89]. Rulings according to international law, however, are by no means a modern phenomenon. As far back as Antiquity, diplomacy and sometimes sanctions were used to regulate the consequences of conquests and military campaigns. Such rules, however, were largely suspended in the era of papal hierocracy and colonialism. Today, international law is mainly aimed at violations of territorial sovereignty, environmental and natural resources, nationality, and the protection of individuals and groups.

Bloody conflicts and wars constantly take place all over the world. They are becoming more frequent and the number of victims continues to grow. In the 20th century almost 200 million people became the victims of wars or armed conflicts and their aftermath[90,91]. In one of the most appalling ethnic massacres in recent history, almost 800,000 people fell victim to genocide in Rwanda, Africa, over a period of just 100 days in 1994[92]. The Global Conflict Tracker of the Council of Foreign Relations (USA) lists 24 violent conflicts worldwide that can be divided into different categories: civil war, criminal violence, interstate conflict, political instability, sectarian violence, territorial dispute, transnational terrorism and unconventional war[93]. Many of these conflicts are motivated by ethnicity, for instance in Nigeria (Christians vs Muslims), where the terrorist militia group Boko Haram is waging war, in Sudan (Dinka vs Nuer or Salva Kiir vs Riek Machar), the Central African Republic, the Congo, Burundi, Somalia and Yemen. The backgrounds to the conflicts are often extremely complex. However, ethnic or social conflicts, be they legitimised by religious differences, social unrest or territorial disputes, are not limited to Africa; they exist all over the world[94]. Shiites and Sunnites are fighting each other in large parts of the Middle East, Turkish forces and the People's Defence Forces (HPG) of the Kurdish Workers' Party (PKK) are engaged in bitter border disputes, as are Israeli and Palestinian forces, whilst the so-called Islamic State group is launching terrorist attacks against nonbelievers all over the world.

In Europe, the breakup of Yugoslavia in the 1990s, caused by a complex mixture of ethnic, religious and economic problems, led to civil war in the Balkan states, which culminated in genocide (Srebrenica)[95]. This first "new" war, as it was called[96], primarily demonstrated that "ethnic nepotism" must be seen "as a cross-cultural background factor of ethnic conflicts"[97]. However, despite large-scale interventions by the global community, true peace has never returned to places like Afghanistan or Iraq. A particularly harrowing situation is currently ongoing in Syria, where the interests of the major powers are actually preventing an end to

the fighting. The conflict between Russia and the Ukraine that is developing in eastern Ukraine, where pro-Russian rebels are fighting Ukrainian forces, is just the latest example of an ethnic conflict motivated by power politics that has not yet been resolved. In this case, it is only as a result of political influence exerted by the US and Europe that the situation has been prevented from worsening.

Text S9. The origin and universality of ethnic nepotism from the perspective of behavioral biology.

The term 'nepotism' denotes a general preferential treatment or protection of family members and relatives over and above unrelated persons in politics, business and many other areas of life[98]. Favouritism towards relatives can go as far as creating familial networks. Nepotism is critically viewed by society, but not necessarily seen in a negative light[99,100]. Ethnic nepotism is an extended form of nepotistic behaviour at group level. This means that people of a particular ethnic background tend to favour people of the same ethnic origin over other ethnic groups. Ethnic conflicts that escalate into violence usually occur in multi-ethnic societies where people of different origin, language and religion live side by side. Throughout the world, conflicts of ethnic interests take place in areas where members of separate ethnic groups meet one another socially and politically, and/or economically compete for the same limited resources[97]. The term 'ethnic nepotism' was coined in the context of African states gaining independence from their colonial powers, often giving rise to tribal rivalries[101].

Scientists initially used evolutionary theses and theories of behavioural biology to explain the origins and universality of nepotism, such as the concept of inclusive fitness and the theory of kin selection as first described by Hamilton[102-104]. The inclusive fitness of an individual is determined by the number of its own genes that are passed on to the next generation. It is composed of the direct fitness, one's own genes in one's own offspring, and the indirect fitness, one's own genes that are passed on to other individuals' offspring via one's relatives. Based on the shared genes between an individual and its relatives, the passing on of its genes by relatives (kin selection) increases the individual's inclusive fitness. W.D. Hamilton devised a rule that explained both the conditions (benefit > cost) under which altruistic behaviour is supported by natural selection and the significance of the degree of kinship. The so-called father of sociobiology, Edward O. Wilson, recently argued against the theory of kin selection[105], prompting much debate. Another theory in the context of ethnic nepotism, the theory of genetic similarity, grew out of altruism research[106]. Not unlike kin selection, this theory argues that an individual is capable of recognising a genetically similar individual and will therefore favour it over less closely related individuals. Applied to human beings, this would mean that the greater the degree of similarity between one individual and another, the more altruistic their behaviour becomes[107]. Whilst ethnic nepotism based on similarity takes a back seat to the role played by social identity, its omnipresence makes it a potential driving force for evolutionary and social change[108].

Text S10. Ethnic identity which promotes inequality between groups is a threat to any society.

The adaptive patterns of behaviour that result in violent clashes between ethnic groups in parallel societies have an evolutionary background and are the subject of numerous theories

about aggression[109]. Whenever ethnic and social identity lead to certain groups becoming isolated from society, violence is the greatest threat to that society and its stability. Isolation has many causes and often harks back to severe social injustice: economic advantage, social dominance and even a lack of empathy. Even the simplest of changes in a group's living circumstances can suffice to make its members perceive a threat to their ethnic identity (e.g. immigration, foreign customs and traditions, increased population density). Evolutionary programmes of behaviour can therefore be activated and violence triggered, even without a threatening situation actually developing between different ethnic groups[110].

Text S11. Key factors for the world of tomorrow.

Genetic diversity is an essential aspect of evolution and therefore of human nature. From a cultural point of view, recent human history appears to have progressed particularly well whenever the powers that be were comfortable with multi-ethnicity and had a tolerance for different denominations, and would even use them as an instrument of governance and integration. This is true both for large empires (Achaemenid Empire, Alexander, Roman Empire, the Huns) and for the nation-states of the 19th/20th centuries (Habsburgs, Russia, Ottoman Empire, Great Britain)[111]. Multiethnicity allows for the broadest diversity possible in many different walks of life, thus presenting competitive advantages over groups with a relatively homogenous configuration. World religions also have their lighter and darker sides. On one hand they are traditionally the key drivers for a life based on cooperation and moral standards; the flip side to this, however, is that they can act out their moral authority, stir up misanthropic prejudice, enact social control, create hierarchy and focus on the maintenance of power[112-114]. Establishing stable social conditions within multiethnic societies in this global world and placing cooperation above competitiveness between religious communities are undoubtedly some of the key positive factors for the world of tomorrow.

References

1 Chesnaux, L. in *Palethnographie du mésolithique. Recherches sur les habitats de plein air entre Loire et Neckar. Actes de la table ronde internationale de Paris, 26 et 27 novembre 2010* (eds Boris Valentin *et al.*) 119-132 (Société préhistorique française, 2013).

2 Gassin, J. *Evolution socio-économique dans le chasséen de la grotte de l'église supérieure (VAR) : apport de l'analyse fonctionnelle des industries lithiques*. (CNRS, 1996).

3 Gibaja, J. F. & Palomo, A. Geométricos usados como proyectiles. Implicaciones económicas, sociales e ideológicas en sociedades neolíticas del VI-III milenio cal BC en el noreste de la Península Ibérica. *Trab Prehist* **61**, 81-97 (2004).

4 Mazzucco, N. *The human occupation of the southern Central Pyrenees in the sixth-third millennia cal BC. A traceological analysis of flaked stone assemblages*. (BAR Publishing, 2018).

5 Elias, N. *The civilizing process*. (Blackwell, 1994).

6 www.economicsandpeace.org (21 March 2019).

7 Parzinger, H. *Die Kinder des Prometheus: Eine Geschichte der Menschheit vor der Erfindung der Schrift*. (Beck, 2015).
8 FAO. *Food and Agriculture Organization of the United Nations. The State of Food and Agriculture 1996*. (United Nations FAO, 1996).
9 Duerr, J. in *Hunting Philosophy for Everyone : in Search of the Wild Life* (ed Nathan Kowalsky) 134-148 (Wiley-Blackwell, 2010).
10 Walker, P. L. A Bioarchaeological Perspective on the History of Violence. *Annu. Rev. Anthrop.* **30**, 573-596 (2001).
11 Wu, X.-J., Schepartz, L. A., Liu, W. & Trinkaus, E. Antemortem trauma and survival in the late Middle Pleistocene human cranium from Maba, South China. *Proc. Natl. Acad. Sci. U. S. A.* **108**, 19558–19562 (2011).
12 Sala, N. *et al.* Lethal interpersonal violence in the Middle Pleistocene. *PLoS One* **10**, e0126589 (2015).
13 Beier, J., Anthes, N., Wahl, J. & Harvati, K. Similar cranial trauma prevalence among Neanderthals and Upper Palaeolithic modern humans. *Nature* **563**, 686-690 (2018).
14 Mirazón Lahr, M. The not-so-dangerous lives of Neanderthals. *Nature* **563**, 634-636 (2018).
15 Allen, M. W. & Jones, T. L. *Violence and warfare among hunter-gatherers*. (Routledge, 2015).
16 Lahr, M. M. *et al.* Inter-group violence among early Holocene hunter-gatherers of West Turkana, Kenya. *Nature* **529**, 394-398 (2016).
17 Orschiedt, J. in *Warfare, violence and slavery in prehistory BAR international series* (eds Michael Parker Pearson & I. J. Thorpe) 67–73 (Archaeopress, 2005).
18 Roksandic, M., Djurić, M., Rakočević, Z. & Seguin, K. Interpersonal violence at Lepenski Vir Mesolithic/Neolithic complex of the Iron Gates Gorge (Serbia-Romania). *Amer. J. Phys. Anthrop.* **129**, 339–348 (2006).
19 Hutton Estabrook, V. in *Violence and warfare among hunter-gatherers* (eds Mark W. Allen & Terry L. Jones) 49–69 (Routledge, 2015).
20 Wahl, J. & Trautmann, I. in *Sticks, stones and broken bones. Neolithic violence in a European perspective* (eds Rick J. Schulting & Linda Fibiger) 77-100 (University Press, 2012).
21 Meyer, C. *et al.* Early Neolithic executions indicated by clustered cranial trauma in the mass grave of Halberstadt. *Nature Communications* **9**, 2472 (2018).
22 Meyer, C., Lohr, C., Gronenborn, D. & Alt, K. W. The massacre mass grave of Schöneck-Kilianstädten reveals new insights into collective violence in Early Neolithic Central Europe. *Proc. Natl. Acad. Sci. U. S. A.* **112**, 11217-11222 (2015).
23 Konopka, T., Szczepanek, A., Przybyła, M. M. & Włodarczak, P. Evidence of interpersonal violence or a special funeral rite in the Neolithic multiple burial from Koszyce in southern Poland: A forensic analysis. *Anthropological Review* **79**, 69–85 (2016).
24 Teschler-Nicola, M. *et al.* Evidence of Genocide 7000 BP: Neolithic Paradigm and Geo-climatic Reality. *Collegium Anthropologicum* **23**, 437–450 (1999).
25 Meller, H. in *3300 BC - Mysteriöse Steinzeittote und ihre Welt* (ed Harald Meller) 239–245 (Landesamt für Archäologie Sachsen-Anhalt, 2013).
26 Otterbein, K. F. *How war began*. (Texas A&M University Press, 2004).
27 Keeley, L. H. *War before civilization*. (Oxford University Press, 1996).

28 Fry, D. P. & Söderberg, P. Lethal Aggression in Mobile Forager Bands and Implications for the Origins of War. *Science* **341**, 270-273 (2013).
29 Martin, D. L., Harrod, R. P. & Pérez, V. R. *The Bioarchaeology of Violence*. (University Press of Florida, 2013).
30 Schulting, R. J. & Fibiger, L. *Sticks, stones and broken bones. Neolithic violence in a European perspective*. (University Press, 2012).
31 Duering, A. & Wahl, J. A massacred village community? Agent-based modelling sheds new light on the demography of the Neolithic mass grave of Talheim. *Anthrop. Anz.* **71**, 447-468 (2014).
32 Boulestin, B. *et al.* Mass cannibalism in the Linear Pottery Culture at Herxheim (Palatinate, Germany). *Antiquity* **83**, 968-982 (2009).
33 Stecher, M., Schlenker, B. & Alt, K. W. in *3300 BC - mysteriöse Steinzeittote und ihre Welt* (ed Harald Meller) 282-289 (Landesamt für Archäologie Sachsen-Anhalt, 2013).
34 Meyer, C. *et al.* The Eulau eulogy: Bioarchaeological interpretation of lethal violence in Corded Ware multiple burials from Saxony-Anhalt, Germany. *J. Anthrop. Archaeol.* **28**, 412-423.
35 Vegas Aramburu, J. I., Armendáriz, Á. & Ajamil, J. *San Juan ante Portam Latinam : una inhumación colectiva prehistórica en el valle medio del Ebro : memoria de las excavaciones arqueológicas, 1985, 1990 y 1991*. (Diputación Foral de Alava, Departamento de Cultura y Euskera, 2007).
36 Guilaine, J. & Zammit, J. *The origins of war: Violence in prehistory*. (Blackwell, 2005).
37 Beyneix, A. Une médecine du fonds des âges: trépanations, amputations et tatouages thérapeutiques au Néolithique. *L'Anthropologie* **119**, 58–71 (2015).
38 Vandkilde, H. in *Warfare and society* (eds Ton Otto, Henrik Thrane, & Helle Vandkilde) 393–422 (Aarhus University Press, 2006).
39 Kristiansen, K. in *Ancient Warfare* (eds John Carman & Anthony Harding) 175–189 (Sutton, 1999).
40 Jantzen, D. *et al.* A Bronze Age battlefield? Weapons and trauma in the Tollense Valley, north-eastern Germany. *Antiquity* **85**, 417-433 (2011).
41 Hobbes, T. *Leviathan, or the matter, forme, and power of a Common-Wealth*. (Andrew Crooke, 1651).
42 Pinker, S. *The better angels of our nature: Why violence has declined*. (Viking, 2011).
43 Münkler, H. in *Frankfurter Zeitung* **18. October** (2011).
44 Welzer, H. *Selbst denken: Eine Anleitung zum Widerstand*. (Fischer, 2015).
45 Gray, J. Delusions of peace. *Prospect. The Leading Magazine of Ideas* **September 21** (2011).
46 Falk, D. & Hildebolt, C. Annual War Deaths in Small-Scale versus State Societies Scale with Population Size Rather than Violence. *Curr. Anthrop.* **58**, 805–813 (2017).
47 Verbeek, B. *Die Wurzeln der Kriege: Zur Evolution ethnischer und religiöser Konflikte*. (Hirzel, 2004).
48 Gómez, J. M., Verdú, M., González-Megías, A. & Méndez, M. The phylogenetic roots of human lethal violence. *Nature* **538**, 233-237 (2016).
49 De Waal, F. *The bonobo and the atheist: In search of humanism among the primates*. (Norton, 2013).
50 Pagel, M. Animal behaviour: Lethal violence deep in the human lineage. *Nature* **538**, 180–181 (2016).

51 Rai, T. S., Valdesolo, P. & Graham, J. Dehumanization increases instrumental violence, but not moral violence. *Proc. Natl. Acad. Sci. U. S. A.* **114** (2017).
52 Saukko, P. & Knight, B. *Knight's Forensic Pathology*. (CRC Press, 2015).
53 Pokines, J. T. & Symes, S. A. *Manual of Forensic Taphonomy*. (CRC Press, 2014).
54 Cohen, H. *et al.* The impact velocity and bone fracture pattern: Forensic perspective. *Forensic Sci. Int.* **266**, 54–62 (2016).
55 Guilaine, J. & Manen, C. From Mesolithic to Early Neolithic in the western Mediterranean. *ProcBritAc* **144**, 21-51 (2007).
56 Redfern, R. C. *Injury and trauma in bioarchaeology: interpreting violence in past lives*. (Cambridge University Press, 2017).
57 Martin, D. L. & Harrod, R. P. Bioarchaeological contributions to the study of violence. *Yb. Phys. Anthrop.* **156**, 116-145 (2015).
58 Junkmanns, J. *Pfeil und Bogen: Von der Altsteinzeit bis zum Mittelalter*. (Hörnig, 2013).
59 Palomo, A. *et al.* Harvesting cereals and other plants in Neolithic Iberia: the assemblage from the lake settlement at La Draga. *Antiquity* **85**, 759-771 (2011).
60 Piqué, R. *et al.* Characterizing prehistoric archery: technical and functional analyses of the Neolithic bows from La Draga (NE Iberian Peninsula). *J. Archaeol. Sci.* **55**, 166-173 (2015).
61 Tarrús, J. La Draga (Banyoles, Catalonia), an Early Neolithic Lakeside Village in Mediterranean Europe. *Catalan Historical Review* **1**, 17–33 (2008).
62 López-Montalvo, E. in *Conflict archaeology Themes in contemporary archaeology* (eds Manuel Fernández-Götz & Nico Roymans) 23–33 (Routledge, 2018).
63 Porcar, J. B. Las pinturas rupestres del Barranco de Les Dogues. *Archivo de Prehistoria Levantina* **4**, 75–80 (1953).
64 van Willigen, S. *Die Neolithisierung im nordwestlichen Mittelmeerraum*. (von Zabern, 2006).
65 Zilhão, J. Radiocarbon evidence for maritime pioneer colonization at the origins of farming in west Mediterranean Europe. *Proc. Natl. Acad. Sci. U. S. A.* **98**, 14180–14185 (2001).
66 Haak, W. *et al.* Massive migration from the steppe was a source for Indo-European languages in Europe. *Nature* **522**, 207-211 (2015).
67 Szécsényi-Nagy, A. *et al.* The maternal genetic make-up of the Iberian Peninsula between the Neolithic and the Early Bronze Age. *Sci. Rep.* **7**, 15644 (2017).
68 Lipson, M. *et al.* Parallel palaeogenomic transects reveal complex genetic history of early European farmers. *Nature* **551**, 368-372 (2017).
69 Brandt, G. *et al.* Ancient DNA Reveals Key Stages in the Formation of Central European Mitochondrial Genetic Diversity. *Science* **342**, 257-261 (2013).
70 Szécsényi-Nagy, A. in *Early farmers* (eds Alasdair W. R. Whittle & Penny Bickle) 71–93 (Oxford University Press, 2014).
71 Groenemeyer, A. in *Die Ethnisierung von Alltagskonflikten* (eds Axel Groenemeyer & Jürgen Mansel) 7–8 (Verlag für Sozialwissenschaften, 2003).
72 Bergmann, W. in *International Handbook of Violence Research* (eds Wilhelm Heitmeyer & John Hagan) 441–460 (Kluwer, 2003).
73 Horowitz, D. L. *The deadly ethnic riot*. (University of California Press, 2001).
74 Janowitz, M. *Social control of escalated riots*. (University of Chicago, 1968).

75 Benz, M. *Die Neolithisierung im Vorderen Orient: Theorien, archäologische Daten und ein ethnologisches Modell*. (Ex oriente, 2000).
76 Bentley, G. R., Paine, R. R. & Boldsen, J. L. in *Reproductive Ecology and Human Evolution* (ed Peter T. Ellison) 203-231 (Aldine de Gruyter, 2001).
77 Bocquet-Appel, J.-P. & Bar-Yosef, O. *The Neolithic Demographic Transition and its Consequences*. (Springer, 2008).
78 Ellison, C. R. W., Chapman, M. R. & Hall, I. R. Surface and deep ocean interactions during the cold climate event 8200 years ago. *Science* **312**, 1929–1932 (2006).
79 Mathieson, I. *et al.* Genome-wide patterns of selection in 230 ancient Eurasians. *Nature* **528**, 499-503 (2015).
80 Szécsényi-Nagy, A. *et al.* Tracing the genetic origin of Europe's first farmers reveals insights into their social organization. *Proceedings of the Royal Society B* **282**, 20150339 (2015).
81 Bramanti, B. *et al.* Genetic Discontinuity between Local Hunter-Gatherers and Central Europe's First Farmers. *Science* **326**, 137-140 (2009).
82 Posth, C. *et al.* Pleistocene Mitochondrial Genomes Suggesta Single Major Dispersal of Non-Africansand a Late Glacial Population Turnover in Europe. *Curr. Biol.* **26**, 827-833 (2016).
83 Fu, Q. *et al.* The genetic history of Ice Age Europe. *Nature* **534**, 200-205 (2016).
84 Meller, H. in *Krieg - eine archäologische Spurensuche* (eds Harald Meller & Michael Schefzik) 19-24 (Landesamt für Denkmalpflege und Archäologie Sachsen-Anhalt, 2015).
85 Bollongino, R. *et al.* 2000 years of parallel societies in Stone Age Central Europe. *Science* **342**, 479–481 (2013).
86 Allentoft, M. E. *et al.* Population genomics of Bronze Age Eurasia. *Nature* **522**, 167-172 (2015).
87 Lazaridis, I. *et al.* Ancient human genomes suggest three ancestral populations for present-day Europeans. *Nature* **513**, 409-413 (2014).
88 Crawford, J. *Brownlie's principles of Public International Law*. (Oxford University Press, 2012).
89 Herdegen, M. *Völkerrecht*. (Beck, 2017).
90 Brzezinski, Z. *Out of Control: Global Turmoil on the Eve of the Twenty-First Century*. Touchstone, 1993).
91 Simons, L. M. Genocide and the Science of Proof. *NaGe* **209**, 28–35 (2006).
92 Gourevitch, P. *We wish to inform you that tomorrow we will be killed with our families: Stories from Rwanda*. (Picador, 1998).
93 www.cfr.org (21 March 2019).
94 Hardin, R. *One for all : the logic of group conflict*. (Princeton University Press, 1995).
95 Bennett, C. *Yugoslavia's bloody collapse: Causes, course and consequences*. (Hurst, 1995).
96 Johnstone, D. *La croisade des fous: Yougoslavie, première guerre de la mondialisation*. (Le Temps des Cerises, 2005).
97 Vanhanen, T. *Global Inequality as a Consequence of Human Diversity: A New Theory Tested by Empirical Evidence*. (Ulster Institute for Social Research, 2014).
98 Hamilton, W. D. in *Kin recognition in animals* (eds David J. C. Fletcher & Charles Duncan Michener) 417–437 (John Wiley & Sons, 1987).
99 Bellow, A. *In praise of nepotism: A natural history*. (Doubleday, 2003).

100 Cashdan, E. Ethnocentrism and Xenophobia: A Cross-Cultural Study. *Curr. Anthrop.* **42**, 760-764 (2001).
101 Van den Berghe, P. L. *The ethnic phenomenon*. (Elsevier, 1981).
102 Hamilton, W. D. The evolution of altruistic behavior. *The American Naturalist* **97**, 354–356 (1963).
103 Hamilton, W. D. The genetical evolution of social behaviour. I. *JTBio* **7**, 1–16 (1964).
104 Hamilton, W. D. The genetical evolution of social behaviour. II. *JTBio* **7**, 17–52 (1964)
105 Wilson, E. O. *The social conquest of earth*. (Norton, 2012).
106 Rushton, J. P., Russell, R. J. & Wells, P. A. Genetic similarity theory: Beyond kin selection. *Behav. Genet.* **14**, 179-193 (1984).
107 Feigin, S., Owens, G. & Goodyear-Smith, F. Theories of human altruism: a systematic review. *Journal of Psychiatry and Brain Functions* **1**, 5 (2014).
108 Salter, F. & Harpending, H. J.P. Rushton's theory of ethnic nepotism. *Pers. Individ. Dif.* **55**, 256-260 (2013).
109 MacDonald, K. An integrative evolutionary perspective on ethnicity. *Politics and the Life Sciences* **20**, 67–80 (2001).
110 Raine, A. *The Anatomy of Violence: The biological roots of crime*. (Random House, 2014).
111 Leonhard, J. & Hirschhausen, U. v. (Vandenhoeck & Ruprecht, Göttingen, 2011).
112 Watts, J., Sheehan, O., Atkinson, Q. D., Bulbulia, J. & Gray, R. D. Ritual human sacrifice promoted and sustained the evolution of stratified societies. *Nature* **532**, 228–231 (2016).
113 Armstrong, K. *Fields of Blood: Religion and the history of violence*. (Doubleday, 2015).
114 Zwingmann, C., Klein, C. & Jeserich, F. *Religiosität: Die dunkle Seite: Beiträge zur empirischen Religionsforschung*. (Waxmann, 2017).
